# Mitochondria directly sense osmotic stress to trigger rapid metabolic remodeling *via* regulation of pyruvate dehydrogenase phosphorylation

**DOI:** 10.1016/j.jbc.2022.102837

**Published:** 2022-12-26

**Authors:** Takeshi Ikizawa, Kazutaka Ikeda, Makoto Arita, Shojiro Kitajima, Tomoyoshi Soga, Hidenori Ichijo, Isao Naguro

**Affiliations:** 1 Laboratory of Cell Signaling, Graduate School of Pharmaceutical Sciences, The University of Tokyo, Tokyo, Japan; 2 Department of Applied Genomics, Laboratory of Biomolecule Analysis, Kazusa DNA Research Institute, Kisarazu, Chiba, Japan; 3 Laboratory of Metabolomics, RIKEN Center for Integrative Medical Sciences, Yokohama, Kanagawa, Japan; 4 Division of Physiological Chemistry and Metabolism, Graduate School of Pharmaceutical Sciences, Keio University, Tokyo, Japan; 5 Institute for Advanced Biosciences, Keio University, Tsuruoka, Yamagata, Japan

**Keywords:** acyl-carnitine, metabolic remodeling, mitochondria, osmotic stress, pyruvate dehydrogenase, 2-DG, 2-deoxyglucose, DCA, dichloroacetate, DMEM, Dulbecco’s modified Eagle’s medium, ECAR, extracellular acidification rate, FAO, fatty acid oxidation, FBS, fetal bovine serum, HK, hexokinase, OCR, oxygen consumption rate, OXPHOS, oxidative phosphorylation, PDH, pyruvate dehydrogenase, PDK, pyruvate dehydrogenase kinase, PDP, pyruvate dehydrogenase phosphatase, PFK, phosphofructokinase, PI, propidium iodide

## Abstract

A high-salt diet significantly impacts various diseases, ilncluding cancer and immune diseases. Recent studies suggest that the high-salt/hyperosmotic environment in the body may alter the chronic properties of cancer and immune cells in the disease context. However, little is known about the acute metabolic changes in hyperosmotic stress. Here, we found that hyperosmotic stress for a few minutes induces Warburg-like metabolic remodeling in HeLa and Raw264.7 cells and suppresses fatty acid oxidation. Regarding Warburg-like remodeling, we determined that the pyruvate dehydrogenase phosphorylation status was altered bidirectionally (high in hyperosmolarity and low in hypoosmolarity) to osmotic stress in isolated mitochondria, suggesting that mitochondria themselves have an acute osmosensing mechanism. Additionally, we demonstrate that Warburg-like remodeling is required for HeLa cells to maintain ATP levels and survive under hyperosmotic conditions. Collectively, our findings suggest that cells exhibit acute metabolic remodeling under osmotic stress *via* the regulation of pyruvate dehydrogenase phosphorylation by direct osmosensing within mitochondria.

In modern society, people are increasingly consuming high-salt diets. High-salt diets are known to have negative effects on human health and exacerbate various diseases, such as hypertension, cardiovascular disease, autoimmune disease, and cancer (1, 2, 3, 4). In particular, many studies have shown a relationship between high-salt diets and cancer, such as the increased risk of gastric cancer and accelerated progression and metastasis of breast cancer by the activation of the MAPK/ERK pathway in cancer cells (5, 6). In contrast, however, there are reports suggesting that feeding a high-salt diet to mice-harboring tumors enhanced antitumor immunity and inhibited tumor progression by suppressing the activity of myeloid-derived suppressor cells (MDSCs) (7, 8). Therefore, the effect of high-salt diets on cancer still remains unclear, and research is necessary to determine how high-salt conditions affect cancer cells and/or surrounding cells and what molecular mechanisms drive these effects.

It has been previously reported that the concentration of sodium ions is increased in tumor tissue (9). For example, there is a higher accumulation of sodium in human breast cancer tissue than in other areas of the breast tissue (10). In addition, an elevated sodium concentration was observed in malignant brain tumors (11). Even though the precise mechanisms of sodium ion accumulation in tumor tissues are not clear, it is suggested that changes in the expression and/or activity of membrane channels and transporters are involved (9). Under chronic hyperosmotic conditions, it has been reported that aerobic glycolysis in cancer cells is enhanced (12). In a breast cancer cell line, elevated sodium increases the expression of enzymes in glycolysis, such as hexokinase (HK) and lactate dehydrogenase, resulting in increased lactate production (13). However, little is known about the acute alteration in glucose and fatty acid metabolism and its underlying mechanisms upon hyperosmotic stress in cancer cells.

Myeloid-derived cells, such as macrophages, infiltrate the tumor microenvironment, and they are known to exhibit plasticity in response to environmental cues. In recent years, it has been suggested that in addition to the kidney, a high-sodium environment is observed in various sites of our body and regulates the immune system, implying that a hyperosmotic environment regulates immunity (14, 15). It has been reported that hyperosmotic conditions promote the differentiation of monocytes into proinflammatory M1 macrophages (16, 17). Furthermore, previous reports suggested in mouse models that a high-salt diet leads to the accumulation of sodium in the tumor and enhances antitumor immunity (7, 8). Immunometabolism has revealed that the metabolic state of immune cells critically affects their polarization and function, including that of tumor-associated macrophages (18, 19, 20). Thus, it is an interesting issue whether the hyperosmotic microenvironment might affect the metabolism of infiltrating immune cells in the tumor.

In the present study, we demonstrate that glucose metabolic remodeling from oxidative phosphorylation (OXPHOS) to aerobic glycolysis (a Warburg-like effect) is rapidly triggered upon hyperosmotic stress both in HeLa cells (a cancer cell line) and in RAW264.7 cells (a mouse monocyte cell line). Moreover, we discovered that fatty acid oxidation (FAO) is acutely suppressed, and acyl-carnitine accumulates under hyperosmotic conditions. We found that phosphorylation of pyruvate dehydrogenase (PDH) is upregulated and downregulated quickly upon hyperosmotic and hypoosmotic stress, respectively, both in cells and in isolated mitochondria. Our results suggest that Warburg-like metabolic remodeling is induced through the rapid regulation of pyruvate dehydrogenase kinase (PDK)-dependent PDH phosphorylation upon hyperosmotic stress, which is directly sensed by mitochondria. Collectively, our results provide new insight into rapid metabolic remodeling upon osmotic stress, which might enable us to regulate cellular metabolism intentionally and rapidly by changing the environmental osmolarity.

## Results

### Warburg-like metabolic remodeling is rapidly induced upon hyperosmotic stress

**Figure 1:**
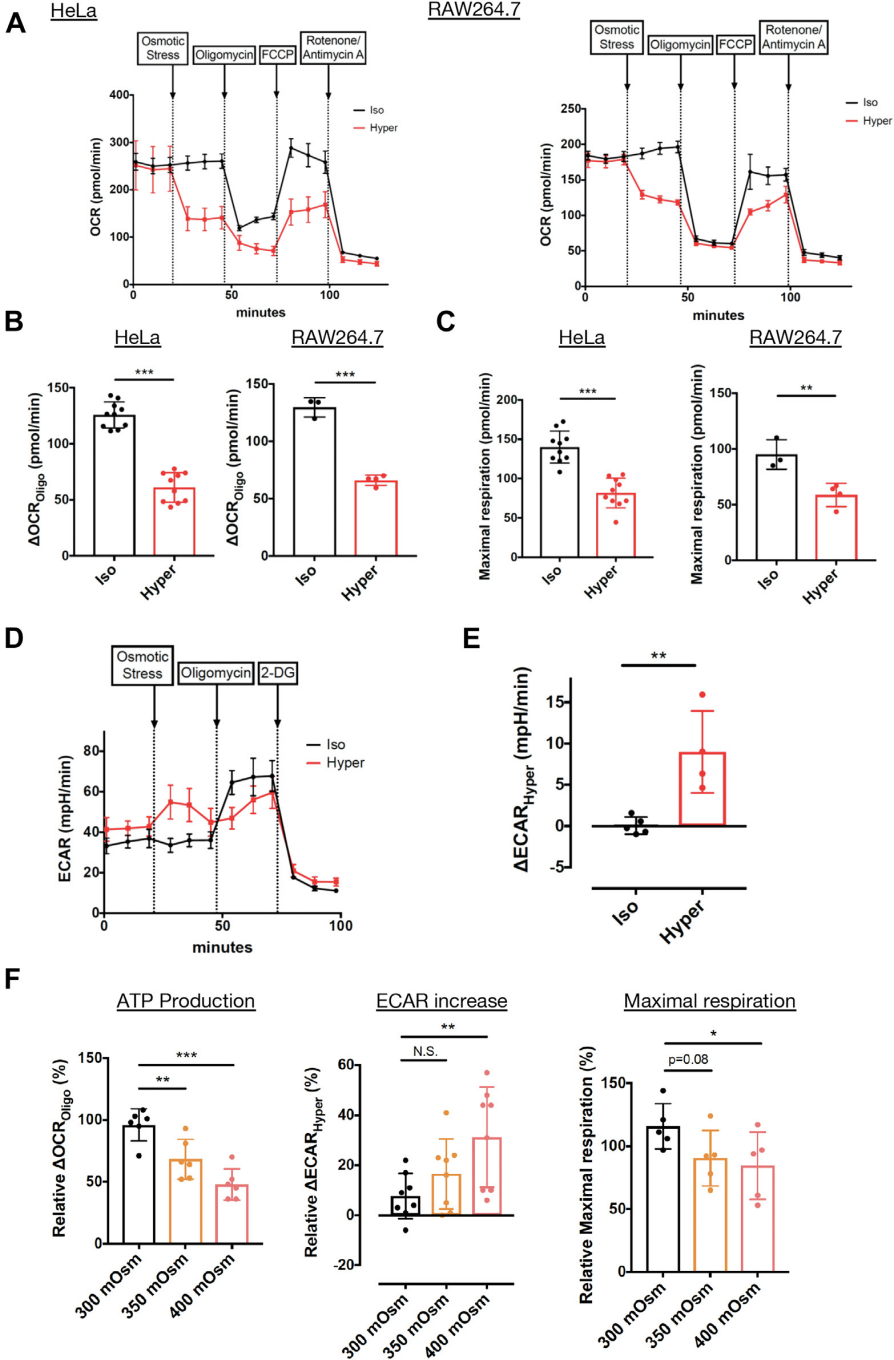
**Acute Warburg-like metabolic remodeling is induced upon hyperosmotic stress.***A*, OCR of HeLa and RAW264.7 cells was measured with sequential treatment with osmotic stress, oligomycin (ATP synthase inhibitor), FCCP (uncoupler), and rotenone (complex I inhibitor)/antimycin A (complex III inhibitor) using an extracellular flux analyzer. A representative result from three independent experiments of each cell line is shown. The trace was the average of 3 to 10 independent wells for each condition. *B*, oligomycin-dependent OCR (ΔOCR_Oligo_) upon osmotic stress extracted from (*A*), which was calculated by subtraction between the average of three sequential measurements before and after oligomycin. *C*, maximal respiration upon osmotic stress extracted from (*A*), which was calculated by subtraction between the average of three sequential measurements before and after FCCP. *D*, ECAR of HeLa cells was measured with sequential treatment with osmotic stress, oligomycin, and 2-DG (hexokinase inhibitor) using an extracellular flux analyzer. A representative result from five independent experiments is shown. The trace was the average of 3 to 5 independent wells for each condition. *E*, increase in ECAR by hyperosmotic stress (ΔECAR_Hyper_) extracted from (*D*), which was calculated by subtraction between the average of three sequential measurements before and after osmotic stress. *F*, relative ΔOCR_Oligo_, ΔECAR_Hyper_, and maximal respiration of HeLa cells under 300, 350, and 400 mOsm. For ΔOCR_Oligo_ and maximal respiration, subtraction between the average of three sequential measurements before osmotic stress and after oligomycin was normalized as 100%. For ΔECAR_Hyper_, the average of the first three sequential measurements was normalized as 100%. See experimental procedures for the detailed calculation method. ΔOCR_Oligo_ (n = 6), ΔECAR_Hyper_ (n = 8), and maximal respiration (n = 5). Isoosmotic stress: 300 mOsm, Hyperosmotic stress: 500 mOsm. Data are represented as the mean ± SD. ∗*p* < 0.05, ∗∗*p* < 0.01, ∗∗∗*p* < 0.001. Unpaired two-tailed Student’s *t* test was conducted for (*B*, *C* and *E*). One-way ANOVA was followed by Dunnett’s multiple comparisons test for (*F*). 2-DG, 2-deoxyglucose; ECAR, extracellular acidification rate; OCR, oxygen consumption rate.

To investigate the rapid change in cellular metabolism upon hyperosmotic stress, we measured the oxygen consumption rate (OCR) and extracellular acidification rate (ECAR), which indicate the rate of mitochondrial respiration and aerobic glycolysis, respectively. We found that OCR markedly decreased upon hyperosmotic stress (500 mOsm) by adding 100 mM sodium chloride to HeLa cells and RAW264.7 cells (Fig. 1*A*). It was a prompt response that could be almost saturated within 10 min (a measurement interval) after stimulation. Oligomycin-sensitive basal OCR (ΔOCR_Oligo_), which is coupled to mitochondrial ATP production, was significantly suppressed upon hyperosmotic stress (Fig. 1*B*). Moreover, maximal respiration, which can be derived from an increase upon FCCP treatment, was also significantly suppressed upon hyperosmotic stress (Fig. 1*C*). We also observed that ECAR rapidly increased upon hyperosmotic stress in HeLa cells (Fig. 1, *D* and *E*), as previously reported (21). As ECAR evaluates protons mainly derived from lactate, the conversion of pyruvate to lactate seemed to be activated upon hyperosmotic stress, similar to the Warburg effect (22). A decrease in OCR and an increase in ECAR (a Warburg-like effect) were also observed by adding 200 mM sorbitol (500 mOsm) as well as sodium chloride stimulation (Fig. S1, *A–E*). These results suggest that the observed Warburg-like effect is triggered by hyperosmolarity but not by a high concentration of sodium chloride itself.

Then, we examined these metabolic changes under milder hyperosmotic stress (Fig. 1*F*). Treatment with 50 mM sodium chloride (400 mOsm) significantly suppressed ΔOCR_Oligo_ and increased ΔECAR_Hyper_. The maximal respiration was also significantly suppressed. In the case of 25 mM sodium chloride (350 mOsm), although the suppression of maximal respiration and enhancement of ECAR were marginal, ΔOCR_Oligo_ was significantly suppressed. Considering that the hyperosmotic environment observed in the human body (except the kidney, exhibiting extremely high osmolarity) reaches up to 400 mOsm (10, 23, 24, 25, 26), the Warburg-like effect observed upon hyperosmotic stress is likely to occur under physiological conditions of various tissues.

As OCR decreased even under relatively mild hyperosmotic stress (350 and 400 mOsm), we next examined how long the OCR decrease is sustained, considering the prolonged exposure to mild hyperosmotic stress may occur in physiological conditions. The decrease in OCR was maintained for 8 h after hyperosmotic stress of 350 and 400 mOsm, suggesting that the acute metabolic remodeling stably persists for at least 8 h (Fig. S1*F*).

As the acute changes in OCR and ECAR were observed dose-dependently by hyperosmotic stress, we further investigated the responses to hypoosmotic stress. Interestingly, OCR significantly increased upon hypoosmotic stress (225 mOsm) (Fig. S1, *G* and *H*). Additionally, OCR suppression by hyperosmolarity was gradually restored to the level of isoosmolarity when we added water to hyperosmotic medium to dilute it to isoosmotic conditions (300 mOsm). Taken together, these results suggest that OCR bidirectionally and flexibly responds to osmotic stress. Meanwhile, ECAR did not change upon hypoosmotic stress (Fig. S1*I*).

### Glucose metabolism shifts toward aerobic glycolysis upon hyperosmotic stress

**Figure 2:**
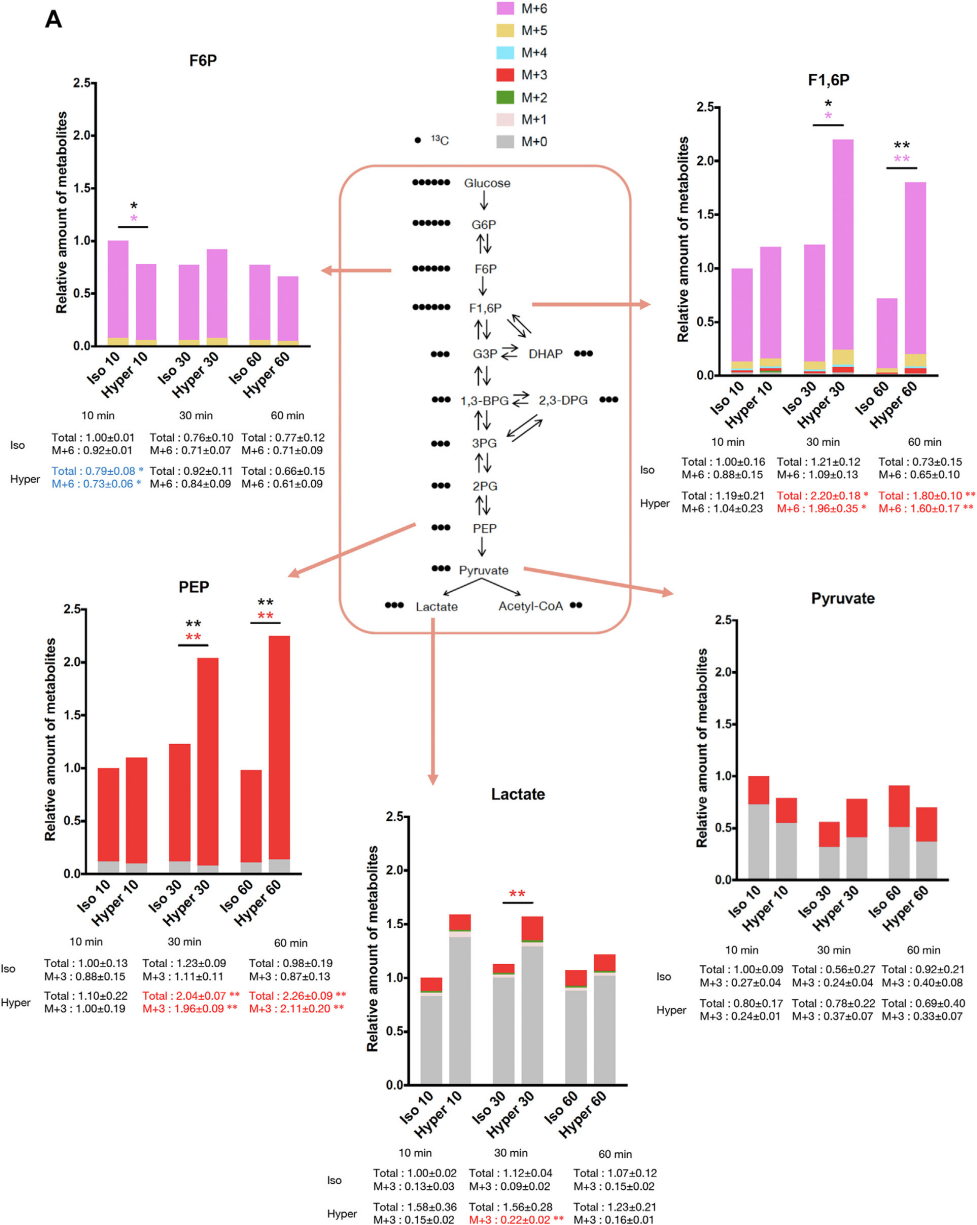

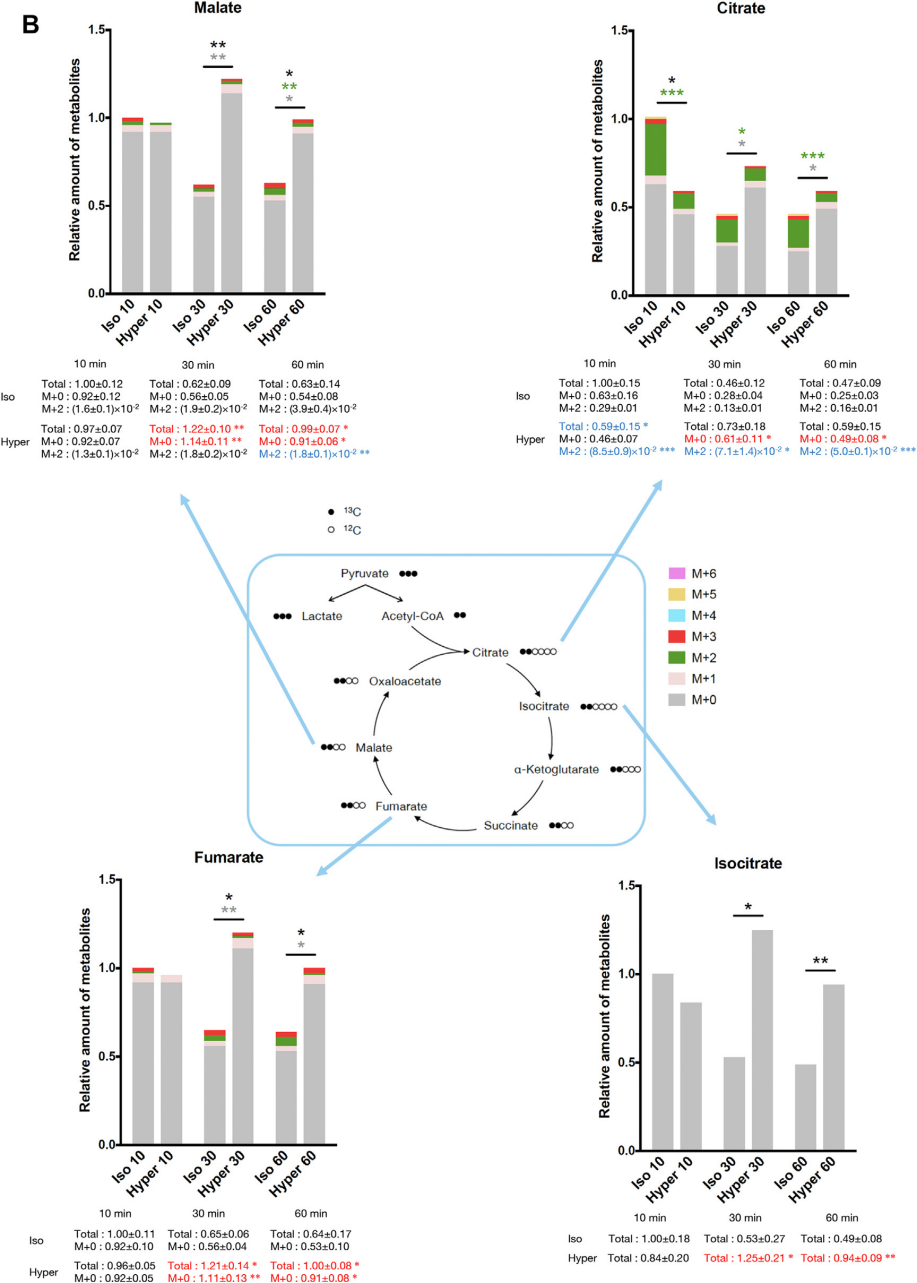

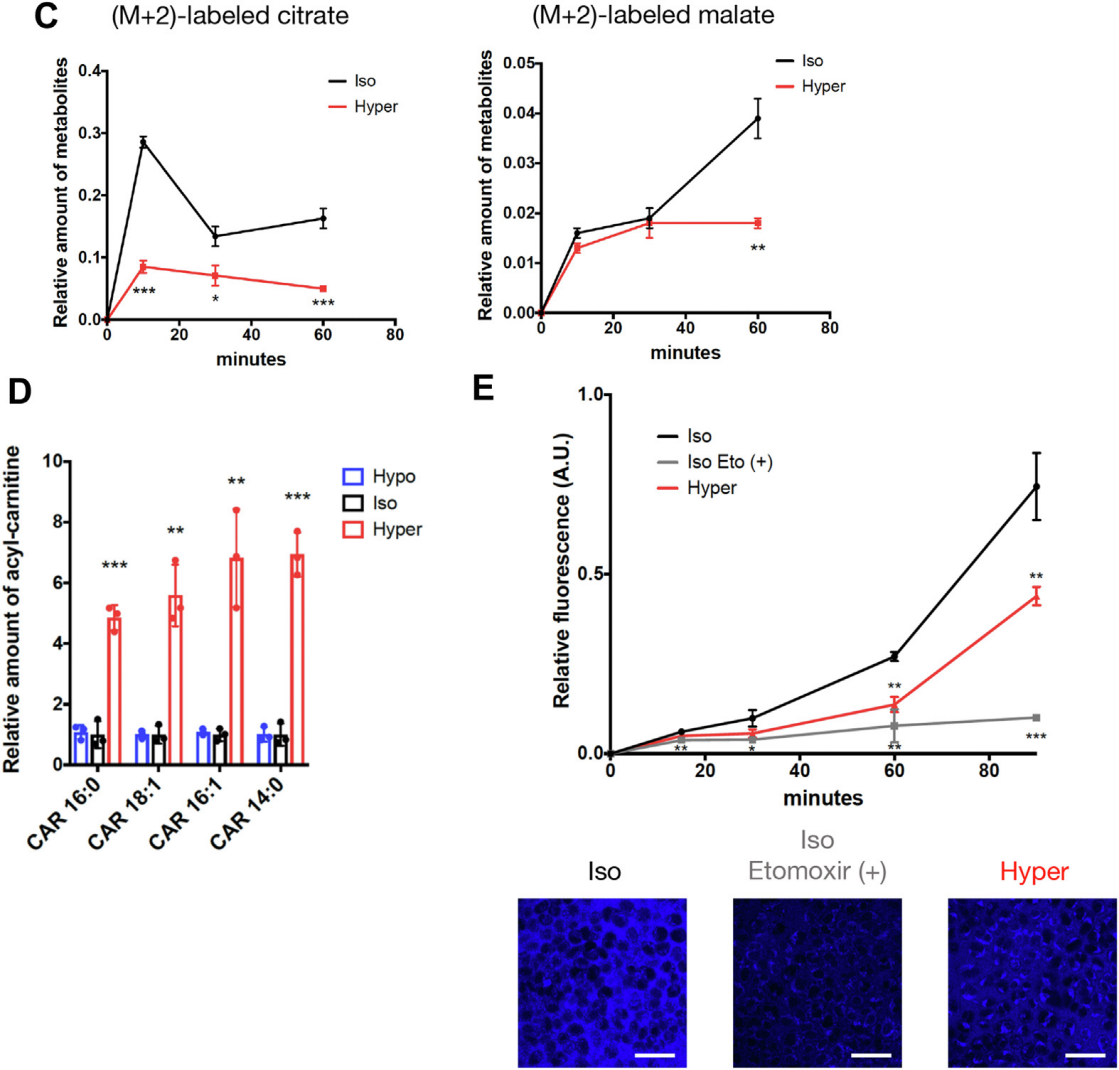
**Aerobic glycolysis is activated and the supply of acetyl-CoA to the TCA cycle is reduced upon hyperosmotic stress.***A* and *B*, metabolome analysis of HeLa cells using ^13^C_6_-glucose tracer under isoosmotic and hyperosmotic conditions for 10, 30, and 60 min. For each metabolite, the relative amounts are normalized by that of the total amount at 10 min under isoosmotic conditions (that is, the total amount at 10 min is 1). The height of each bar graph depicts the relative amount of a metabolite in total (nonlabeled to full-labeled). The color codes of each ^13^C-labeled metabolite are indicated in top of the Figure. The relative amounts of several metabolites labeled with a specific number of ^13^C are shown by text under each bar graph. Metabolites significantly increased and decreased under hyperosmotic conditions are shown in *red* and *blue* letters, respectively. Statistics were performed by comparing isoosmotic and hyperosmotic conditions at the same timepoint. The color of ∗ corresponds to the color of each labeled body. The ∗ in *black* indicates a difference in the total amount of the metabolite. Metabolites in glycolysis (F6P, F1,6P, PEP, pyruvate, and lactate) are shown in (*A*), and metabolites in the TCA cycle (citrate, isocitrate, fumarate, and malate) are shown in (*B*). All other metabolites are shown in Table S1. *C*, line graphs of the relative amount of (M + 2)-labeled citrate and malate extracted from (*B*). *D*, relative amount of acyl-carnitines (composed of 16:0, 18:1,16:1, and 14:0 fatty acids) detected by nontargeted lipidome analysis. Samples were harvested from HeLa cells after 15 min of osmotic stress. The amount of each acyl-carnitine (CAR n:n) is normalized to that in isoosmotic conditions. *E*, fluorescence intensity of FAOBlue in HeLa cells at 15, 30, 60, and 90 min after osmotic stress. Three independent experiments were performed in triplicate from independent wells. The *upper line* graph shows a representative result with the mean ± SD of triplicate experiments. Etomoxir (40 μM) was pretreated before osmotic stress for 30 min. The *lower panels* show FAOBlue fluorescence images of HeLa cells at 90 min after osmotic stress. Etomoxir (40 μM) was pretreated before osmotic stress for 60 min. Scale bar represents 50 μm. Hypoosmotic stress: 200 mOsm, Isoosmotic stress: 300 mOsm, Hyperosmotic stress: 500 mOsm. Data are represented as the mean ± SD. N = 3 (three samples for each condition) except (*E*). ∗*p* < 0.05, ∗∗*p* < 0.01, ∗∗∗*p* < 0.001. Unpaired two-tailed Student’s *t* test was conducted at the same time points for (*A–C*). One-way ANOVA was followed by Dunnett’s multiple comparisons test for (*D* and *E*). F1,6P, fructose 1,6-bisphosphate; F6P, fructose 6-phosphate; PEP, phosphoenolpyruvate.

To examine the contribution of glucose metabolites to the Warburg-like effect upon hyperosmotic stress, we conducted a metabolome analysis using a fully labeled ^13^C_6_-glucose tracer (Table S1). Among the detected metabolites in glycolysis, metabolites toward phosphoenolpyruvate were mostly replaced with the full-labeled form within 10 min in both isoosmotic and hyperosmotic conditions (Fig. 2*A*). The amount of full-labeled fructose 1,6-bisphosphate was significantly increased under hyperosmotic conditions compared with isoosmotic conditions at 30 and 60 min, although there was no change in the amount of full-labeled fructose 6-phosphate (Fig. 2*A*). These data suggest that phosphofructokinase (PFK) might be activated under hyperosmotic stress. Moreover, the total amount and full-labeled form of intermediate metabolites in glycolysis, dihydroxyacetone phosphate, 2,3-diphosphoglycerate, 3-phosphoglycerate, and phosphoenolpyruvate, were also increased under hyperosmotic conditions compared with isoosmotic conditions (Figs. 2*A* and S2*A*). Finally, the end product of aerobic glycolysis, lactate, showed an increasing tendency in total amount and a significant increase in full-labeled form at 30 min in hyperosmotic conditions, although pyruvate did not significantly increase in total amount and full-labeled form, suggesting that the conversion of pyruvate to lactate was activated upon hyperosmotic stress. This is consistent with the increase in ECAR upon hyperosmotic stress (Fig. 1, *D* and *E*).

Regarding the metabolites in the TCA cycle, the amount of (M + 2)-labeled citrate (containing two ^13^C), which is mainly derived from acetyl-CoA through glycolysis, was significantly suppressed upon hyperosmotic stress (Fig. 2, *B* and *C*). The amount of (M + 2)-labeled malate was also suppressed under hyperosmotic conditions at 60 min. Although we could not precisely measure acetyl-CoA in the metabolome analysis, defects in ^13^C incorporation into TCA cycle metabolites suggest that the supply of glucose-derived acetyl-CoA to the TCA cycle might be reduced under hyperosmotic conditions.

On the other hand, nonlabeled (M + 0) citrate, isocitrate, fumarate, and malate significantly accumulated at 30 and 60 min under hyperosmotic stress compared with isoosmotic conditions (Fig. 2*B*). This result may reflect the previous observation that OXPHOS is directly impaired by hyperosmotic stress (17), resulting in a decrease in the rate of the TCA cycle. On the other hand, it is also possible that resources of acetyl-CoA other than glucose might expand in hyperosmotic conditions to increase nonlabeled metabolites in the TCA cycle. As fatty acids are another main source of acetyl-CoA (27), we comprehensively examined lipid dynamics in osmotic conditions by a nontargeted lipidome analysis (28). We found that various species of acyl-carnitine, an intermediate metabolite of fatty acid metabolism in mitochondria (β-oxidation), markedly increased at 15 min of hyperosmotic stress (Fig. 2*D*). There was no difference in the amount of any kind of triglycerides, which are sources of acyl-carnitine (Fig. S2*B* and Table S2). Then, we quantified the FAO activity in HeLa cells upon osmotic stress using FAOBlue, the fluorescence of which reflects the β-oxidation activity in live cells (29). FAOBlue fluorescence intensity, which was abolished by etomoxir (an inhibitor of carnitine palmitoyl transferase 1), was attenuated under hyperosmotic stress (Fig. 2*E*). Furthermore, in culture medium with rich palmitic acid and no glucose, the maximal respiration (depending on fatty acid metabolism) was suppressed under hyperosmotic stress (Fig. S2*C*). Collectively, these results indicate that β-oxidation was suppressed by hyperosmotic stress, in parallel with the accumulation of acyl-carnitine. Therefore, it is conceivable that acetyl-CoA derived from fatty acids was also decreased under hyperosmotic conditions, suggesting that the accumulation of nonlabeled metabolites in the TCA cycle (seen in Fig. 2*B*) was due to the reduction in consumption in the TCA cycle.

### PDH is suppressed through phosphorylation by PDK upon hyperosmotic stress, which is directly sensed by mitochondria

**Figure 3:**
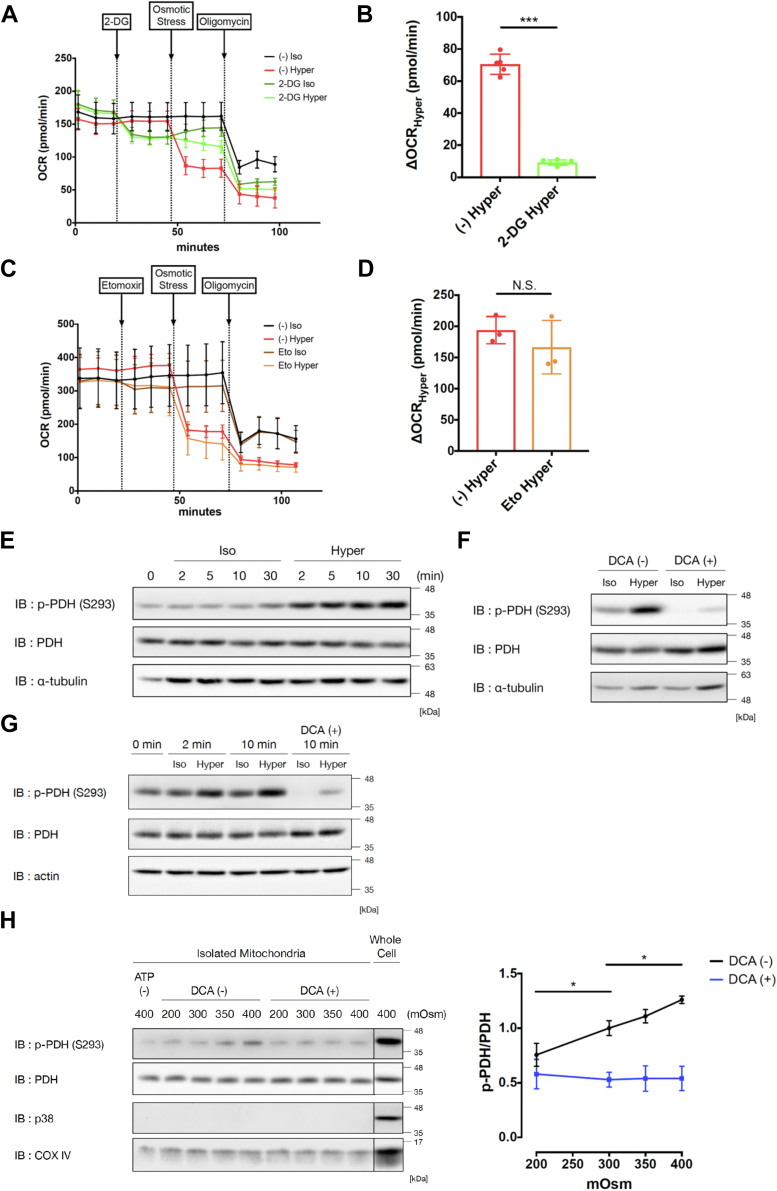
**PDK-dependent PDH phosphorylation is induced in mitochondria upon hyperosmotic stress.***A* and *C*, OCR of HeLa cells with sequential treatment with 2-DG (30 mM) (*A*) or etomoxir (20 μM) (*C*), osmotic stress, and oligomycin. A representative result from three independent experiments is shown. The trace was the average of 3 to 7 (*A*) or 3 to 10 (*C*) independent wells for each condition. *B* and *D*, decrease in OCR by hyperosmotic stress (ΔOCR_Hyper_) extracted from (*A*) or (*C*), respectively, which was calculated by subtraction between the average of three sequential measurements before and after osmotic stress. *E*, immunoblot analysis of PDH phosphorylation after 2, 5, 10, and 30 min of osmotic stress in HeLa cells. *F*, immunoblot analysis of PDH phosphorylation after 10 min of osmotic stress with and without pretreatment with DCA (25 mM) for 60 min in HeLa cells. *G*, immunoblot analysis of PDH phosphorylation after 2 and 10 min of osmotic stress in RAW264.7 cells. DCA (25 mM) was pretreated for 60 min. *H*, immunoblot analysis of PDH phosphorylation in isolated mitochondria from HeLa cells. Isolated mitochondria were directly exposed to 10 min of osmotic stress (200, 300, 350, and 400 mOsm). DCA (25 mM) was pretreated for 30 min. ATP (500 nM) was added at the same time as osmotic stimulation. Whole-cell samples of HeLa cells were prepared after 30 min of hyperosmotic stimulation (400 mOsm). The right graph shows the ratio of p-PDH to PDH quantified from the immunoblot. Three independent experiments were performed for quantification of the band intensities. Isoosmotic stress: 300 mOsm, Hyperosmotic stress: 500 mOsm. Data are represented as the mean ± SD. ∗*p* < 0.05, ∗∗∗*p* < 0.001. Unpaired two-tailed Student’s *t* test was conducted for (*B*), (*D*). One-way ANOVA was followed by Dunnett’s multiple comparisons test for (*H*). 2-DG, 2-deoxyglucose; DCA, dichloroacetate; PDH, pyruvate dehydrogenase; PDK, pyruvate dehydrogenase kinase; OCR, oxygen consumption rate.

The decrease in OCR upon hyperosmotic stress was attributable to a reduction in acetyl-CoA supply (from glucose and fatty acids) and/or defects in the electron transport chain (17). First, we evaluated the contribution of acetyl-CoA supply from glucose and fatty acid metabolism to the decrease in OCR under normal culture conditions. Surprisingly, prior inhibition of glycolysis by 2-deoxy-glucose (2-DG) strongly abrogated the decrease in OCR upon hyperosmotic stress, suggesting that the alteration in glycolysis is the main reason for the acute decrease in OCR (Fig. 3, *A* and *B*). Considering that OCR was almost maintained even under hyperosmotic stress in 2-DG conditions, in contrast to a previous report regarding mononuclear phagocytes (17), a direct defect in the electron transport chain is not the main reason for the acute OCR decrease upon hyperosmotic stress in the present context. In contrast, prior inhibition of β-oxidation by etomoxir treatment scarcely abolished the decrease in OCR induced by hyperosmotic stress (Fig. 3, *C* and *D*). These results suggest that under normal culture conditions, suppression of acetyl-CoA supply *via* glycolysis plays a pivotal role in acute OCR suppression upon hyperosmotic stress.

Then, we hypothesized that PDH, which converts pyruvate to acetyl-CoA, might be inactivated under hyperosmotic stress, similar to the general Warburg effect (30). PDH is inactivated in various conditions, such as hypoxia or nutrient starvation, by the phosphorylation of serine residues S232, S293, or S300 (31). Among these phosphorylation sites, S293 is reported to be phosphorylated the earliest and its phosphorylation has the highest effect on PDH inhibition (32, 33). Thus, we detected S293 phosphorylation to see the response of PDH after osmotic stress. We found that the phosphorylation status of PDH(S293) was upregulated upon hyperosmotic stress (Fig. 3*E*). The upregulation was observed as early as 2 min and was sustained until 30 min upon hyperosmotic stimulation. This result suggests that PDH inactivation may suppress the acetyl-CoA supply from pyruvate, resulting in the activation of lactate production. PDH(S293) phosphorylation was also upregulated upon hyperosmotic stress by adding sorbitol, confirming the importance of osmolarity itself (Fig. S3*A*). In addition, the upregulation of PDH after 350 and 400 mOsm of hyperosmotic stress was sustained for at least 8 h (Fig. S3*B*), which is consistent with the persistence of OCR decrease (Fig. S1*F*).

Interestingly, corresponding to the OCR increase under hypoosmotic stress (Fig. S1*E*), PDH(S293) phosphorylation decreased upon hypoosmotic stress (Fig. S3*C*). The phosphorylation status of PDH(S293) was gradually altered in both directions in an osmolarity-dependent manner. Moreover, PDH(S293) phosphorylation was flexibly reversed after inversing osmotic stress (Fig. S3*D*). These results suggest that PDH(S293) phosphorylation is regulated bidirectionally and reversibly under osmotic stress.

PDH(S293) is phosphorylated by PDK (31). Pretreatment with dichloroacetate (DCA), a PDK inhibitor, strongly suppressed PDH(S293) phosphorylation upon hyperosmotic stress, suggesting that PDK is responsible for the phosphorylation of PDH(S293) under hyperosmotic stress (Fig. 3*F*). Upregulation of PDH(S293) phosphorylation upon hyperosmotic stress and its dependence on PDK were also observed in RAW264.7 cells (Fig. 3*G*).

Pyruvate dehydrogenase phosphatase (PDP) is a phosphatase which dephosphorylates PDH at Ser293 (32). It is possible that PDP inactivation may be involved in the upregulation of PDH phosphorylation after hyperosmotic stress. As PDP is activated by Ca^2+^ increase in mitochondria (32, 34), we examined whether mitochondrial Ca^2+^ concentration might be decreased after hyperosmotic stress using a mitochondria Ca^2+^ probe (Rhod-2 AM). Ca^2+^ concentration in mitochondria was not decreased under both iso- and hyper-osmotic stress until 30 min after stimulation (Fig. S3*E*), suggesting PDK activation but not PDP suppression may contribute to PDH phosphorylation under hyperosmotic stress.

Next, we examined the involvement of ERK, AMPK, and Akt, which were previously reported to regulate PDK with their kinase activities (35, 36, 37, 38, 39). However, an MEK inhibitor (U0126) did not abrogate the phosphorylation of PDH(S293) upon hyperosmotic stress (Fig. S3*F*). The mechanism underlying the regulation of PDK by AMPK and Akt is unclear, and both positive and negative regulation have been reported (36, 37, 38, 39). AMPK activity monitored by S79 phosphorylation of acetyl-CoA carboxylase (Fig. S3*G*) and Akt activity monitored by S473 phosphorylation of Akt (Fig. S3*H*) were downregulated under hyperosmotic stress, implying that negative regulation of PDK by AMPK or Akt (36, 38), if any, could be applicable under hyperosmotic conditions. However, AMPK activator (AICAR) did not suppress the phosphorylation of PDH(S293) upon hyperosmotic stress, although the phosphorylation of acetyl-CoA carboxylase was sustained by the treatment. The result suggests that negative regulation of PDK by AMPK does not contribute to the phosphorylation of PDH(S293) upon hyperosmotic stress (Fig. S3*G*). In addition, an Akt inhibitor (MK-2206) did not upregulate the phosphorylation of PDH(S293) under basal conditions, implying that Akt may not negatively regulate PDK in HeLa cells (Fig. S3*H*).

Some metabolites, such as ADP and NAD^+^, are endogenous inhibitors of the PDK activity (40, 41). Thus, we further examined whether the concentrations of ADP or NAD^+^ might be reduced after hyperosmotic stress. However, there was no change in both ADP and NAD^+^ concentrations under hyperosmolarity until 30 min (Fig. S3, *I* and *J*), suggesting PDK activity is not altered by the change in these metabolites.

Both PDH and PDK are localized in mitochondria (42). It has been reported that mitochondrial respiration activity is related to their own volume change (43). Considering together that the cytoplasmic kinases (ERK, AMPK, and Akt) upstream of PDK did not contribute to the regulation of PDH(S293) phosphorylation upon hyperosmotic stress, we next examined whether direct application of osmotic stress to mitochondria may alter the phosphorylation status of PDH(S293). We isolated mitochondria from HeLa cells and directly treated them with osmotic stress. The phosphorylation status of PDH was altered bidirectionally to osmotic stress and was inhibited by pretreatment with DCA (Fig. 3*H*). These results suggest that mitochondria themselves can directly sense osmotic stress and change PDK activity. Furthermore, because no substrate for the TCA cycle was included in the reaction buffer, it is suggested that the alteration in PDK activity is independent of the secondary effect through changes in metabolites involved in glycolysis and the TCA cycle.

### Warburg-like metabolic remodeling contributes to the maintenance of ATP amount and survival under hyperosmotic stress

**Figure 4:**
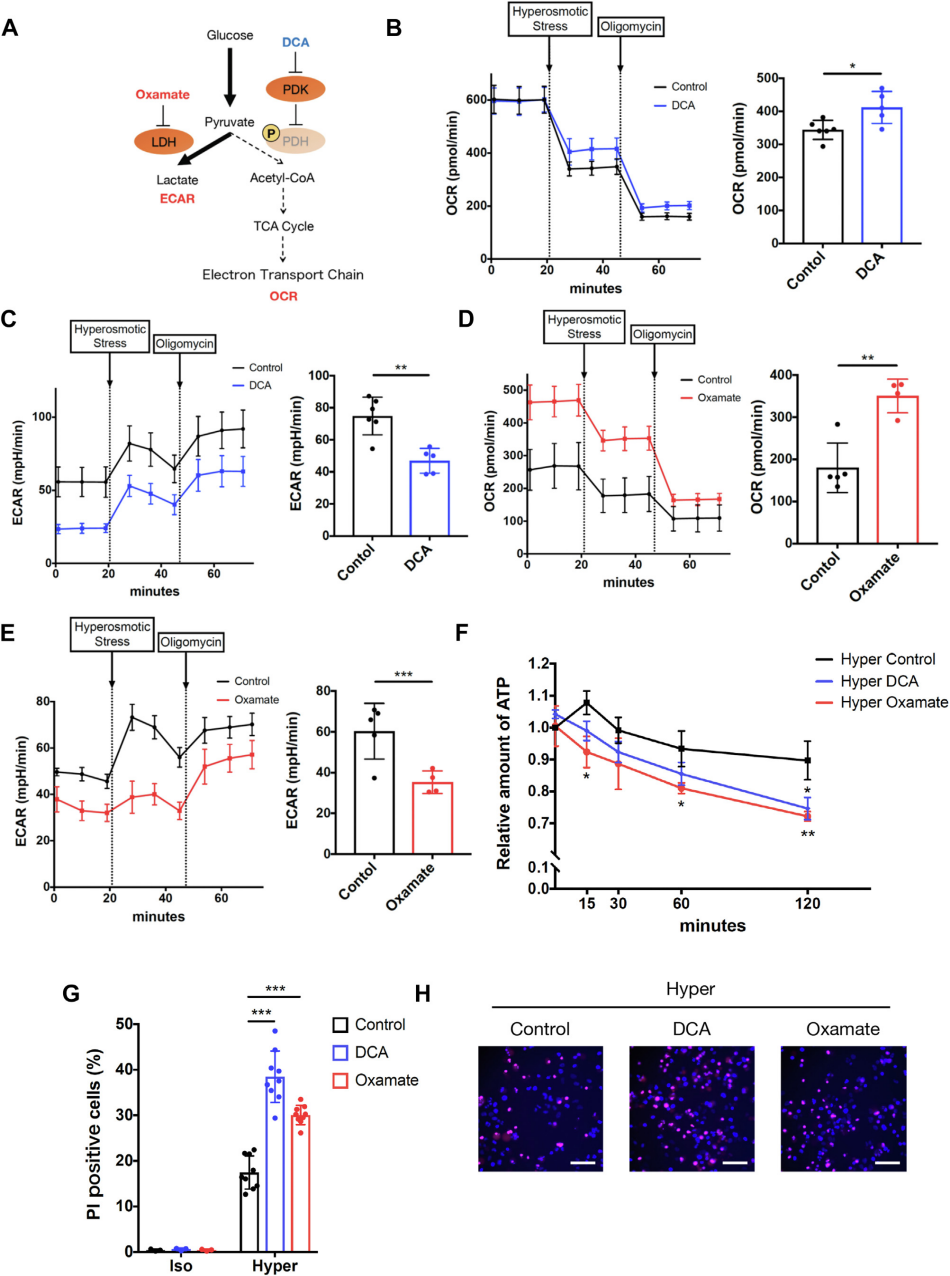

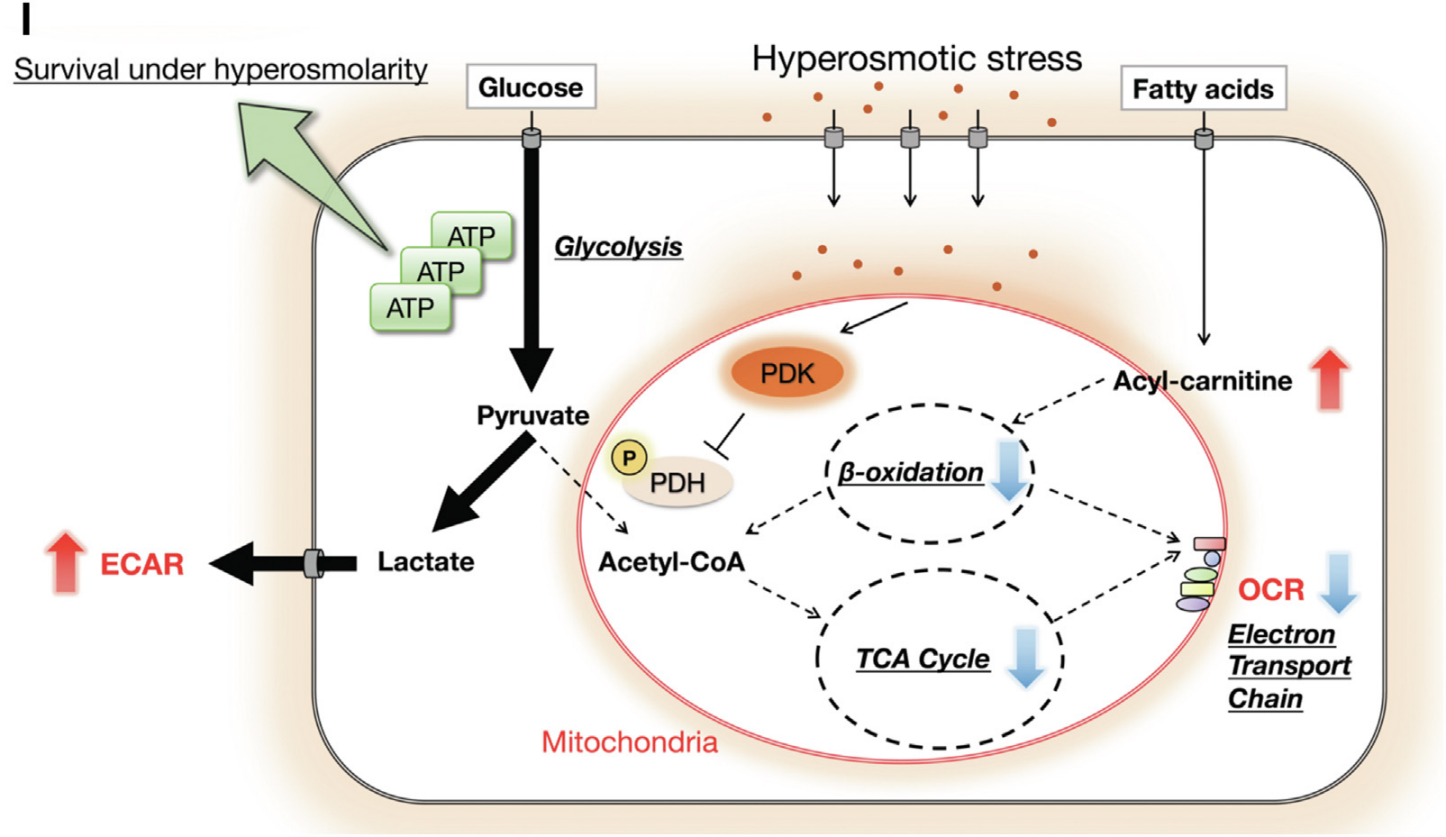
**Warburg-like metabolic remodeling contributes to the maintenance of ATP levels and survival under hyperosmotic stress.***A*, a schematic model of metabolic remodeling in hyperosmotic stress and molecular targets of DCA and oxamate. *B*, OCR and (*C*) ECAR of HeLa cells upon hyperosmotic stress with pretreatment with NaCl (37.5 mM) or DCA (50 mM) for 30 min before the assay. NaCl was pretreated in the control to equalize the osmolarity with DCA treatment. A representative result from three independent experiments is shown. The trace was the average of 3 to 6 independent wells for each condition. Bar graphs show OCR (*B*) and ECAR (*C*) upon hyperosmotic stress, which was calculated by averaging the three sequential measurements upon hyperosmotic stress. *D*, OCR and (*E*) ECAR of HeLa cells upon hyperosmotic stress with pretreatment with NaCl (37.5 mM) or oxamate (50 mM) for 30 min before the assay. NaCl was pretreated in the control to equalize the osmolarity with oxamate treatment. A representative result from three independent experiments is shown. The trace was the average of 3 to 6 independent wells for each condition. Bar graphs show OCR (*D*) and ECAR (*E*) upon hyperosmotic stress, which was calculated by averaging the three sequential measurements upon hyperosmotic stress. *F*, The relative amount of ATP in HeLa cells was measured upon hyperosmotic stress with pretreatment with DCA (25 mM) or oxamate (25 mM) for 60 min using a plate reader. The timepoint of osmotic stimulation was defined as 0 min. Three independent experiments were performed and averaged. *G* and *H*, percentage of PI-positive HeLa cells after 24 h of osmotic stress with pretreatment with DCA (25 mM) or oxamate (25 mM) for 60 min measured by an image analyzer. Three independent experiments were performed and averaged in (*G*). Representative images of PI-stained HeLa cells under hyperosmotic conditions are shown in (*H*). Scale bar represents 100 μm. *I*, a schematic model of cellular metabolic changes upon hyperosmotic stress. When cells are subjected to hyperosmotic stress, the cytosol becomes hyperosmotic because of water efflux, resulting in an osmotic gradient between the cytosol and mitochondria. The osmotic change is directly sensed by the mitochondria and activates PDK, which phosphorylates PDH and suppresses its activity. This causes a shift in glucose metabolism from OXPHOS to aerobic glycolysis, resulting in a decrease in OCR and an increase in ECAR. Activation of aerobic glycolysis produces ATP quickly, which may contribute to cell survival under hyperosmotic conditions. Furthermore, fatty acid metabolism in mitochondria (β-oxidation) is suppressed, and acyl-carnitine accumulates upon hyperosmotic stress, which may be involved in the decrease in OCR. Isoosmotic stress: 300 mOsm, Hyperosmotic stress: 500 mOsm. Data are represented as the mean ± SD. ∗*p* < 0.05, ∗∗*p* < 0.01, ∗∗∗*p* < 0.001. Unpaired two-tailed Student’s *t* test was conducted for (*B–E*). One-way ANOVA was followed by Dunnett’s multiple comparisons test for (*F* and *G*). DCA, dichloroacetate; ECAR, extracellular acidification rate; OCR, oxygen consumption rate; OXPHOS, oxidative phosphorylation; PDH, pyruvate dehydrogenase; PDK, pyruvate dehydrogenase kinase; PI, propidium iodide.

To investigate the physiological meaning of rapid metabolic remodeling upon hyperosmotic stress, we focused on the cellular ATP level, assuming that the shift from OXPHOS to aerobic glycolysis may produce ATP in a faster manner (44). We used DCA and oxamate (lactate dehydrogenase inhibitor) to suppress the Warburg-like metabolic remodeling observed under hyperosmotic stress. DCA suppresses the step of inhibitory phosphorylation of PDH, while oxamate suppresses the step of pyruvate to lactate conversion (Fig. 4*A*). Both inhibitors actually suppressed the Warburg-like metabolic remodeling under hyperosmotic stress. Pretreatment with DCA inhibited the decrease in OCR upon hyperosmotic stress, resulting in a higher OCR under hyperosmotic conditions (Fig. 4*B*). The ECAR was decreased in the basal state with DCA treatment and remained lower than that of the control upon hyperosmotic stress (Fig. 4*C*). Oxamate increased the basal OCR, and the OCR remained higher than that of the control upon hyperosmotic stress (Fig. 4*D*). The increase in ECAR upon hyperosmotic stress was inhibited by pretreatment with oxamate, resulting in a lower ECAR under hyperosmotic conditions (Fig. 4*E*). These results suggest that both inhibitors can be used as suppressors of the Warburg-like remodeling (lower OCR and higher ECAR) upon hyperosmotic stress.

Then, we investigated the effects of these inhibitors on the amount of cellular ATP. Pretreatment with DCA or oxamate did not affect ATP levels under isoosmotic conditions (Fig. S4). In contrast, the relative ATP level upon hyperosmotic stress was significantly decreased by pretreatment with these inhibitors compared with the control (Fig. 4*F*). Considering that the production rate of ATP is approximately 100 times faster in glycolysis than in OXPHOS (44), the result suggests that Warburg-like remodeling upon hyperosmotic stress is induced to compensate for ATP demand under stress conditions by rapidly producing ATP in glycolysis (9).

Finally, we examined the impact of the inhibition of metabolic remodeling on cell death under hyperosmotic conditions. Cell death evaluated by propidium iodide (PI) staining was not detectable under isoosmotic conditions even in the presence of DCA or oxamate (Fig. 4*G*). However, PI-positive cells were significantly increased by pretreatment with DCA or oxamate under hyperosmotic stress (Fig. 4, *G* and *H*). These data suggest that activation of aerobic glycolysis is necessary for maintaining the ATP level and cell survival under hyperosmotic conditions.

## Discussion

In the present study, we demonstrated that Warburg-like metabolic remodeling occurs within a few minutes upon hyperosmotic stress in HeLa and RAW264.7 cells, in which glucose metabolism shifts to aerobic glycolysis from OXPHOS (Fig. 4*I*). We also demonstrated that FAO activity is suppressed under hyperosmotic conditions (Fig. 2*E*). Glucose metabolic remodeling is induced at least in part by the phosphorylation and inactivation of PDH by PDK (Fig. 3, *F* and *G*). Treatment with 2-DG abolished the decrease in OCR upon hyperosmotic stress (Fig. 3, *A* and *B*), suggesting that hyperosmotic stress affects glucose metabolism to suppress OCR but not affects the electron transport chain directly as previously reported (17, 43). Although there have been reports suggesting that hyperosmotic stress enhances aerobic glycolysis (13) and suppresses mitochondrial respiration (17, 45) after several hours or days, we report in this study that immediate phosphorylation of PDH upon hyperosmotic stress is an acute trigger of glucose metabolic remodeling in cancer and macrophage cell lines. We also confirmed that the decrease in OCR and upregulation of PDH(S293) phosphorylation were maintained until at least 8 h after mild hyperosmotic stress (Figs. S1*F* and S3*B*), suggesting that the acute remodeling in glucose metabolism persists for several hours under mild hyperosmotic stress.

We have not revealed the detailed molecular mechanisms by which PDK is regulated in osmotic stress, even though we investigated several candidates of signaling pathways that have been reported to be upstream of PDK and also metabolites (ADP and NAD^+^) which inhibit PDK activity (Fig. S3, *F–J*). On the other hand, we found that phosphorylation of PDH in isolated mitochondria is also altered bidirectionally to osmotic stress, suggesting that the mitochondria themselves directly sense osmolarity to change PDK activity (Fig. 3*H*). Although it has been suggested that mitochondrial respiration activity has some relation to their volume change, the precise molecular mechanisms were unknown (43). Our findings provide a new insight that the PDK–PDH axis might be involved in the volume sensing under osmotic stress. Cells exposed to osmotic stress undergo volume changes along with alterations in the morphology and tension of the cellular membrane by swelling or shrinking (46). It is possible that the properties of the mitochondrial membrane might also change under osmotic stress and affect PDK activity. A previous report showed in yeast that the activity of target of rapamycin complex 2 is regulated by plasma membrane tension under osmotic stress (47). A similar mechanism might govern PDK activity on the mitochondrial membrane. Further studies are required to elucidate the sensing mechanism of osmolarity in mitochondria to alter PDK activity.

It has been reported that the expression of enzymes responsible for irreversible steps in glycolysis (HK, PFK, and pyruvate kinase) is increased in cancer cells, which is a hallmark of the Warburg effect (48). As mentioned in the results section, PFK might be activated upon hyperosmotic stress (Fig. 2*A*). In addition, even though the total amount of fructose 1,6-bisphosphate was markedly increased, the total amount of G6P and fructose 6-phosphate did not decrease significantly (Fig. 2*A* and Table S1), suggesting a sufficient supply of G6P from glucose by the activation of HK. Therefore, the activation of aerobic glycolysis under hyperosmotic conditions may be derived not only from PDH suppression but also from the activation of enzymes such as PFK and HK, such as the Warburg effect (48). However, the activation mechanism of these enzymes in hyperosmotic stress is considered to be different from the general Warburg effect in terms of speed. In the general Warburg effect, transcription factors such as hypoxia inducible factor-1 and cellular myelocytomatocis are activated to promote the transcription of PDK, which contributes to the enhanced phosphorylation of PDH (49). The metabolic remodeling in this study is a fast response that occurs in a few minutes, implying that it is triggered by quick signal transduction rather than regulation of gene expression, which takes a relatively long time to be achieved.

We also demonstrated that fatty acid metabolism (β-oxidation) is suppressed upon hyperosmotic stress with accumulation of acyl-carnitine (Fig. 2, *D* and *E*), suggesting that it contributes to the suppression of OCR as well as glucose metabolic remodeling through the decrease of acetyl-CoA supply. However, it is still unclear whether the accumulation of acyl-carnitine is the result of the suppression of β-oxidation. The defect in β-oxidation would simply result in the accumulation of a substrate, acyl-carnitine. It is also conceivable that impairment of acyl-carnitine transport to the mitochondrial matrix caused by defects in carnitine acyl-carnitine translocase and/or carnitine palmitoyl transferase 2 may abrogate β-oxidation. Thus, the molecular mechanism by which hyperosmotic stress increases acyl-carnitine in the cells and the physiological meaning of the inhibition of fatty acid metabolism have yet to be elucidated.

The results presented in our study demonstrate that glucose metabolic remodeling upon hyperosmotic stress is induced to produce ATP in a faster manner by activating aerobic glycolysis and has a pivotal role in cell survival under hyperosmotic conditions (Fig. 4). Under isoosmotic conditions, pretreatment with DCA or oxamate did not affect ATP levels, suggesting that ATP produced by aerobic glycolysis could be compensated (Fig. S4). Without any inhibitors, there was almost no difference in the amount of ATP under hyperosmotic conditions compared with isoosmotic conditions (Figs. 4*F* and S4). This result suggests that ATP is consumed as rapidly as ATP is produced by aerobic glycolysis under hyperosmotic stress. It has been reported that hyperosmotic stress causes DNA damage (50) and that ATP is required for DNA repair (51). Therefore, it is possible that ATP is consumed for the repair of DNA damage upon hyperosmotic stress.

In addition to HeLa cells, we demonstrated that glucose metabolic remodeling in hyperosmotic stress occurs in RAW264.7 cells (Figs. 1*A* and 3*G*). Activating aerobic glycolysis is one of the important remodeling pathways for macrophages to promote polarization into proinflammatory M1 macrophages (20). In addition, recent studies have shown that differentiation into M1 macrophages is enhanced under hyperosmotic conditions (16). As described earlier, the tumor microenvironment is suggested to be hyperosmotic (9), and a high-salt diet is reported to result in further high-sodium conditions in tumors and inhibit tumor growth (7). Although the report focused on myeloid-derived suppressor cells, it is possible that the enhanced differentiation into M1 macrophages by activating aerobic glycolysis under such hyperosmotic conditions may contribute to suppressing cancer progression by showing their antitumor activities. Furthermore, inflammation and bacterial infection sites in the body are also reported to be hyperosmotic environments (26, 52). It is assumed that immune cells that migrated to these sites would also be subjected to rapid hyperosmotic stress. Differentiation into M1 macrophages through metabolic remodeling might contribute to the inflammatory response at these sites. In addition, metabolic remodeling may be necessary for macrophage survival in affected areas with hyperosmolarity.

In this study, we found that the OCR and phosphorylation status of PDH were bidirectionally and flexibly altered by osmotic stress (Figs. S1*G* and S3, *C* and *D*). Because OCR was restored when the hyperosmolarity was returned to isoosmolarity by adding water (Fig. S1*G*), it is proposed that the decrease in OCR upon hyperosmotic stress is not a defective but a controlled and reversible change with flexible plasticity. These results suggest that the balance of glucose metabolism between OXPHOS and aerobic glycolysis can be intentionally and immediately controlled by changing the osmolarity in the tumor, which is expected to provide a way to control the metabolic state of cancer and immune cells in cancer therapy.

In summary, our findings revealed rapid metabolic remodeling upon osmotic stress through PDK-dependent PDH phosphorylation. Elucidation of the acute regulatory mechanism of PDK in mitochondria will provide a novel methodology to control cellular metabolism without altering osmolarity. Although it should be clarified whether and, if any, which cells exhibit osmotic stress-dependent metabolic remodeling *in vivo*, further studies will provide new insights into the rapid regulation of metabolism by osmolarity, which might contribute to the treatment of cancer, immune diseases, and other metabolic diseases.

## Experimental procedures

### Cell culture

HeLa cells were cultured in Dulbecco’s modified Eagle’s medium (DMEM)-low glucose (Sigma, Cat#D6046 or Wako, Cat#041-29775) supplemented with 10% fetal bovine serum (FBS). RAW264.7 cells were cultured in RPMI-1640 medium (Sigma, Cat#R8758 or Wako, Cat#189-02025) supplemented with 10% FBS. All cells were cultured in 5% CO_2_ at 37 °C.

### Osmotic stress treatments

Osmolality of the medium is approximately 300 mOsm. 2.5 M NaCl (Wako, Cat#195-01663) solution was added to the medium at final concentrations of 25 mM, 50 mM, and 100 mM, resulting in hyperosmotic stress of approximately 350 mOsm, 400 mOsm, and 500 mOsm, respectively. By adding 2.5 M sorbitol (Wako; Cat#198-03755) solution to the medium at a final concentration of 200 mM, a hyperosmotic stress of approximately 500 mOsm was applied. Hypoosmotic stress was applied by adding distilled water (MilliQ) in the medium to the osmolality of 200 mOsm, 225 mOsm, and 250 mOsm. Absolute osmolality was verified by Osmomat 030 (Gonotec).

### Antibodies

The following antibodies were used in this study: rabbit monoclonal anti-phospho-PDH-E1α (Ser293) antibody (EPR12200: Abcam, Cat#ab177461), mouse monoclonal anti-PDH-E1α antibody (D-6: Santa Cruz Biotechnology, Cat#sc-377092), rat monoclonal anti-α-tubulin antibody (YL1/2: Santa Cruz Biotechnology, Cat#sc-53029), mouse monoclonal anti-actin antibody (AC-40: Sigma, Cat#A3853), mouse monoclonal anti-p38α MAPK antibody (L53F8: Cell Signaling, Cat#9228), rabbit polyclonal anti-COXⅣ antibody (proteintech, Cat#11242-1-AP), mouse monoclonal anti-phospho-p44/42 MAPK (Erk1/2) (Thr202/Tyr404) antibody (E10: Cell signaling, Cat#9106), rabbit polyclonal anti-p44/42 MAPK (Erk1/2) antibody (Cell Signaling, Cat#9102), rabbit polyclonal anti-phospho-acetyl-CoA-carboxylase (Ser79) antibody (Cell Signaling, Cat#3661), rabbit monoclonal anti-acetyl-CoA-carboxylase antibody (C83B10: Cell Signaling, Cat#3676), rabbit polyclonal anti-phospho-Akt (Ser473) antibody (Cell Signaling, Cat#9271), rabbit monoclonal anti-Akt antibody (11E7: Cell Signaling, Cat#4685), and rabbit polyclonal anti-phospho-4E-BP1 (Ser65) antibody (Cell Signaling, Cat#9451). Goat anti-rabbit IgG, HRP-linked antibody (Cell Signaling, Cat#7074), horse anti-mouse IgG, HRP-linked antibody (Cell Signaling, Cat#7076), and goat anti-rat IgG, HRP-linked antibody (Cell Signaling, Cat#7077) were used as secondary antibodies.

### Measurement of OCR and ECAR

OCR and ECAR measurements were performed using the XF24 or XF96 Extracellular Flux analyzer (Seahorse Bioscience). Cells were plated into XF24 (Agilent; Cat#100867-100) or XF96 (Agilent; Cat#102601-100) cell culture plates and cultured for 24 h. Prior to performing the assay, culture medium in the wells was exchanged with the Seahorse XF DMEM medium (Agilent, Cat#103575-100) containing 1 g/l glucose (Wako, Cat#041-00595), 2 mM glutamine (Sigma, Cat#G9273), and 1 mM sodium pyruvate (Sigma, Cat#P2256) for HeLa cells or Seahorse XF RPMI medium (Agilent, Cat#103576-100) containing 2 g/l glucose and 1 mM glutamine for RAW264.7 cells. While sensor cartridges were calibrated, cell plates were incubated in a 37 °C, CO_2_–free incubator for 60 min prior to the start of the assay. All experiments were performed at 37 °C. Each measurement cycle consisted of a mixing time of 3 min, a waiting time of 2 min, and a data acquisition time of 3 min. Timepoints of OCR and ECAR data refer to the average rates during the measurements cycle. All compounds were prepared at appropriate concentrations in desired assay medium. In a typical experiment, three baseline measurements were taken prior to the addition of any compound, and three response measurements were taken after the addition of each compound. The OCR and ECAR data were analyzed using Wave software (Agilent; https://www.agilent.com/en/product/cell-analysis/real-time-cell-metabolic-analysis/xf-software/seahorse-wave-desktop-software-740897).

For the experiment of measuring maximal respiration dependent on fatty acid metabolism (Fig. S2*C*), HeLa cells were seeded in XF96 cell culture plate. One day after seeding, culture medium in the wells was exchanged with DMEM medium (Sigma, Cat#D5030) containing 0.5 mM glucose, 1 mM glutamine, 0.5 mM carnitine (Sigma, Cat#C0283), 1% FBS, 15 mg/l phenol red (Wako, Cat#165-01121), and 3.7 g/l NaHCO_3_ (Nacalai Tesque, Cat#312-13). Next day, prior to performing the assay, the medium was exchanged with assay medium (111 mM NaCl, 4.7 mM KCl, 1.25 mM CaCl_2_, 2.0 mM MgSO_4_, 1.2 mM NaH_2_PO_4_, 2.5 mM glucose, 0.5 mM carnitine, and 5 mM Hepes). One hundred fifty micromolar palmitate-bovine serum albumin (Agilent, Cat#102720-100) was added just before starting the assay. Forty micromolar (+)-etomoxir (sodium salt) (Cayman Chemical, Cat#11969), if present, was added 15 min before starting the assay.

For the experiment of measuring OCR and ECAR under 350 and 400 mOsm (Fig. 1*F*), each value was calculated as follows. Relative ΔOCR_Oligo_ = 100∗(average of three sequential measurements after osmotic stress − average of three sequential measurements after oligomycin)/(average of the first three sequential measurements − average of three sequential measurements after oligomycin). Relative maximal respiration = 100∗(average of three sequential measurements after FCCP − average of three sequential measurements after oligomycin)/(average of the first three sequential measurements − average of three sequential measurements after oligomycin). Relative ΔECAR_Hyper_ = 100∗(average of three sequential measurements after osmotic stress − average of the first three sequential measurements)/average of the first three sequential measurements.

For the experiment of measuring OCR until 8 h (Fig. S1*F*), three baseline measurements were taken prior to osmotic stress, and 65 response measurements were taken after osmotic stress.

The reagents pretreated 30 min before the assay include: 50 mM sodium DCA (Sigma, Cat#347795), 50 mM sodium oxamate (Wako, Cat#327-24621). The reagents treated for some indicated experiments include 3 to 5 μM oligomycin A (Sigma, Cat# 75351), 1 μM (for HeLa cells) or 0.6 μM (for RAW264.7 cells) carbonyl cyanide *p*-trifluoromethoxyphenylhydrazone (FCCP) (Cayman, Cat#15218), 2 μM rotenone (Sigma, Cat#R8875), 4 μM antimycin A (Sigma, Cat#A8674), 30 mM 2-deoxy-glucose (Tokyo Kasei, Cat#D0051), and 20 μM (+)-etomoxir (sodium salt). Reagents were injected from the reagent ports automatically to the wells at the time as indicated in the figures.

### Metabolome analysis using a fully labeled ^13^C_6_-glucose tracer

HeLa cells were seeded at 7 × 10^5^ cells on a 10-cm dish and cultured overnight. Three dishes (triplicates) were prepared for each condition. Next day, culture media were replaced with 8 ml of ^13^C_6_ glucose-containing culture media. Osmotic stress was started at the same time by adding 0.5 ml of 150 mM (for 300 mOsm) and 1900 mM (for 500 mOsm) sodium chloride solution for isoosmotic stress and hyperosmotic stress, respectively. After 10, 30, and 60 min of osmotic stress, cells were washed twice with 5% mannitol solution and lysed in 1 ml methanol containing 25 μM internal standards (L-methionine sulfone, 2-(N-morpholino) ethanesulfonic acid, and D-camphor-10-sulfonic acid). After incubating for 10 min at room temperature, the cells and supernatants were collected and stored at −80 °C until analysis.

Charged metabolites were extracted from 400 μl lysate with 400 μl of chloroform and 200 μl of Milli-Q water, passing 400 μl of the aqueous phase through a 5 kDa-cutoff spin-filter column. The filtrate was dried using an evacuated centrifuge and resuspended in 25 μl Milli-Q water containing 200 μM reference compounds (3-aminopyrrolidine and Trimesate) prior to analysis by MS.

All the charged metabolites in samples were measured by CE-TOFMS (Agilent Technologies). Detailed conditions for CE-TOFMS–based metabolome analysis were described previously (53). For CE-TOFMS system control and data acquisition, we used our proprietary software MasterHands (agilent.com).

For data analysis, the amount of each metabolite was normalized by the average of the total amount at 10 min under isoosmotic conditions. Then, the triplicates in each stimulation condition were averaged and compared. Metabolites exhibiting the coefficient of variation of triplicate less than 0.4 in all six conditions (3 timepoints in iso- and hyper-osmotic conditions each) were used for statistics. Graphs were drawn by using Prism software (https://www.graphpad.com/scientific-software/prism/).

### Nontargeted lipidome analysis

HeLa cells were seeded at 1.4 × 10^6^ cells on a 10-cm dish. Three dishes (triplicates) were prepared for each condition (200 mOsm hypoosmotic, 300 mOsm isoosmotic, and 500 mOsm hyperosmotic stimulation). The background control dishes contained only medium. After two days, the dishes were washed twice with PBS and then replaced with each osmotic buffer adjusted by mannitol. Isoosmotic buffer (300 mOsm) contained 130 mM NaCl, 2 mM KCl, 1 mM KH_2_PO_4_, 2 mM CaCl_2_, 2 mM MgCl_2_, 10 mM Hepes, 10 mM glucose, and 20 mM mannitol. Hyperosmotic buffer (500 mOsm) contained additional 200 mM mannitol compared with isoosmotic buffer. In hypoosmotic buffer (200 mOsm), mannitol was excluded and NaCl was reduced to 90 mM from isoosmotic buffer. The dishes were incubated at room temperature for 15 min and the buffer was aspired. Six milliliters of cold MeOH were added to each dish at 4 °C. After incubating for 10 min at 4 °C, the supernatants were collected and stored at −80 °C until analysis.

The supernatants were transferred to glass jacket tubes (FCR&Bio). Then, they were concentrated by centrifugation (Labconco) overnight at 4 °C. Hundred microliters of chloroform were added to dried cells, followed by 30 s sonication. After 60 min incubation at room temperature, 200 μl of methanol containing internal standards was added and vortexed for 10 s. After 60 min of incubation, 20 μl of Milli-Q water was added, vortexed for 10 s, and incubated for 10 min at room temperature. The tubes were then centrifuged at 2000*g* for 10 min at 20 °C, and 200 μl of supernatant was transferred to LC-MS vials. The final composition ratio of the lipid extracts was chloroform: methanol: water (5:10:1, v/v/v), and the final concentrations of internal standards were prepared to 25 μM for fatty acids (16:0-d3, 18:0-d3) and 5 μM for acyl-carnitine (18:0-d3).

Identification and quantification of lipid-related molecules were performed using LC-MS/MS as described previously with some modifications (28, 54). Briefly, 1 μl of lipid extracts was separated by an ACQUITY UPLC BEH C18 column (2.1 × 50 mm, particle size 1.7 μm) at a flow rate of 300 μl/min at 45 °C using an ACQUITY UPLC system (Waters). Solvent A consisted of acetonitrile/methanol/water (20:20:60, v/v/v) and solvent B was isopropanol, both containing 5 mM ammonium acetate and 10 nM EDTA. The solvent composition started at 100% (A) for the first 1 min and was changed linearly to 64% (B) at 7.5 min, where it was held for 4.5 min. The gradient was increased linearly to 82.5% (B) at 12.5 min, followed by 85% (B) at 19 min, 95% (B) at 20 min, 100% (A) at 20.1 min, and 100% (A) at 25 min.

Analysis of lipid-related molecules was performed using a Triple TOF 6600 system (AB SCIEX) in the negative and positive ion mode with a scan range of *m/z* 70 to 1250. Raw data files from the TOF-MS were converted to MGF files using the program AB SCIEX converter for subsequent quantitative analysis with 2DICAL (Mitsui Knowledge Industry). Identification of molecular species was accomplished by comparison with retention times and MS/MS spectra with commercially available standards or reference samples.

For data analysis, the amount of each lipid-related molecule was normalized by the average of isoosmotic conditions. Then, the triplicates in each osmotic condition were averaged and compared.

### Live-cell imaging and measurement of FAOBlue fluorescence

For live-cell imaging experiment, HeLa cells were seeded in φ35 mm glass bottom dishes (Matsunami, Cat#D11130H) which were coated with 1% gelatin (Nacalai Tesque, Cat#16605-42) in PBS in advance. Two days later, the culture medium was aspired and washed with FBS (-) culture medium. Then, 1 ml of culture medium containing 20 μM FAOBlue (Funakoshi, Cat#FDV-0033) was added per dish and osmotic stimulation was simultaneously applied. Forty micromolar etomoxir, if present, was pretreated 60 min before osmotic stimulation. Ninety minutes after osmotic stimulation, the dish was imaged by a TCS SP5 (Leica) confocal-laser scanning microscope equipped with a stage top incubator (Tokai Hit). The cells were observed in 5% CO_2_ at 37 °C using an HC PL APO 63 × /1.40 oil objective (Leica). FAOBlue was excited at 405 nm. Representative images were adjusted linearly to appropriate brightness using Image J software (https://imagej.nih.gov/ij/).

For measuring the fluorescence intensity of FAOBlue, HeLa cells were seeded in a 96-well black glass bottom plate (Matsunami, Cat#GP96000) which were coated with 1% gelatin in PBS in advance. Two days later, the culture medium was aspired and washed with FBS (-) culture medium. Then, 100 μl of culture medium containing 20 μM FAOBlue was added per well and osmotic stimulation was simultaneously applied. Forty micromolar etomoxir, if present, was pretreated 30 min before osmotic stimulation. Osmotic stress was treated for 15, 30, 60, and 90 min. The fluorescence of FAOBlue was measured at the wavelength (Ex/Em = 405/460 nm) using Varioskan Flash (Thermo Fisher Scientific).

### Immunoblotting

Cells or isolated mitochondria were lysed in lysis buffer (20 mM Tris–HCl pH 7.5, 150 mM NaCl, 10 mM EDTA pH 8.0, 1% sodium deoxycholate, 1% Triton X-100, 1 mM PMSF, and 5 μg/ml leupeptin) for 20 min at 4 °C. The extracts were clarified by centrifugation for 10 min, and the supernatants were sampled by adding an equal volume of 2 × SDS sample buffer (80 mM Tris–HCl pH 8.8, 80 μg/ml bromophenol blue, 28.8% glycerol, 4 % SDS, and 10 mM DTT). After boiling at 98 °C for 3 min, the samples were resolved by SDS-PAGE and electroblotted onto a FluoroTrans W membrane (Pall, Cat#BSP0161) or an Immobilon-P membrane (Millipore, Cat#IPVH00010). The membranes were blocked with 5% skim milk (Megmilk Snow Brand) in TBS-T (50 mM Tris–HCl pH 8.0, 150 mM NaCl, and 0.05% Tween 20) and then probed with the appropriate primary antibodies diluted by 1st antibody-dilution buffer (TBS-T supplemented with 5% BSA (Iwai Chemicals, Cat#A001) and 0.1% NaN_3_ (Nacalai Tesque, Cat#312-33)). After replacing and probing the appropriate secondary HRP-conjugated antibodies diluted in 5 % skim milk in TBS-T, antibody–antigen complexes were detected by FUSION SOLO S (VILBER) using an ECL system (GE Healthcare). Quantification was performed *via* densitometry using Fusion Capt software (VILBER; https://www.garvan.org.au/research/capabilities/molecular-genetics/documents/fusion_manual_2016.pdf). Representative images were adjusted linearly to the appropriate brightness and contrast using Fusion Capt software (VILBER).

### Measurement of mitochondrial Ca^2+^ concentration

HeLa cells were seeded in a 96-well black plate (Porvair Sciences, Cat#205003) and cultured for 24 h. Two micromolar Rhod-2 AM (Abcam, Cat#ab142780) and 0.02% Pluronic F-127 (Invitrogen, Cat#P3000MP) diluted in HBSS were pretreated 75 min before osmotic stress. After 60 min, cells were washed with HBSS, and 200 μM MnCl_2_ diluted in DMEM-low glucose was treated. After 15 min, just before stimulating osmotic stress, the fluorescence of Rhod-2 AM was measured (time 0) at the wavelength (Ex/Em = 552/581 nm) using Varioskan Flash. Then, iso- or hyper-osmotic stress was applied. The fluorescence of Rhod-2 AM was measured at 5, 15, 30 min after osmotic stress. The ratiometric fluorescent signal at each timepoint (F) was normalized by average fluorescent signal at time 0 (F_0_), to show relative mitochondrial Ca^2+^ level.

### Measurement of intracellular ADP concentration

Intracellular ADP concentration was measured using ADP/ATP Ratio Assay Kit-Luminescence (DOJINDO, Cat#346-09911). HeLa cells were seeded in a 96-well white transparent bottom plate (Greiner Bio-One, Cat#655098) and cultured for 24 h. Iso- or hyper-osmotic stress was applied for 5, 15, 30 min. After osmotic stimulation, ATP working solution was added to each well. Samples were incubated at room temperature for 10 min protected from light, and the luminescence was measured using Varioskan Flash. Then, ADP working solution was added to each well and incubated at room temperature for 8 min protected from light. Wells without adding ADP working solution were also prepared. The luminescence was measured and the subtraction between the wells with and without ADP working solution was calculated (=fluorescence intensity proportional to ADP concentration). ADP concentration was calculated by the standard curve.

### Measurement of intracellular NAD^+^ concentration

Intracellular NAD^+^ concentration was measured using NAD/NADH-Glo Assay (Promega, Cat#G9071). HeLa cells were seeded in a 96-well plate and cultured for 24 h. Iso- or hyper-osmotic stress was applied for 5, 15, 30 min. After osmotic stimulation, NAD^+^ and NADH was separately detected following the manufacture’s protocol. NAD^+^ concentration was calculated by the standard curve.

### Mitochondria isolation

HeLa cells were collected from a confluent 15-cm dish and centrifuged. The supernatant was discarded, and the precipitated cells were suspended with buffer A (270 mM Mannitol, 10 mM Hepes/K^+^ pH 7.4, 0.2 mM EDTA/K^+^ pH 8.0, and 0.1% BSA). The centrifugation and resuspension procedures were repeated at least twice to completely replace the culture medium with buffer A. Then, the cells were disrupted using a homogenizer and centrifuged at 800*g* for 10 min at 4 °C. The supernatant was decanted into another tube, centrifuged at 3000*g* for 8 min at 4 °C, followed by continuous centrifugation at 7600*g* for 5 min at 4 °C. After discarding the supernatant, 10 ml of buffer B (270 mM mannitol, 10 mM Hepes/K^+^ pH 7.4, and 0.1% BSA) was added, and the precipitates were suspended and centrifuged at 800*g* for 10 min at 4 °C. The supernatant was decanted into another tube and centrifuged at 6700*g* for 10 min at 4 °C. Finally, 2.8 ml of buffer B was added to the precipitate (isolated mitochondria) and suspended. Of note, the experimental instruments in the isolation were immersed in buffer A beforehand to remove Ca^2+^, and all procedures were performed on ice.

After centrifugating the mitochondrial suspension and discarding the supernatant, osmotic buffer was added to the precipitate and suspended. ATP (500 nM) was added at the same time as osmotic simulation. 200 mOsm osmotic buffer contained 90 mM KCl, 10 mM Hepes/K^+^ pH 7.4, 0.2 mM EDTA/K^+^ pH 8.0, and 0.1% BSA. 300 mOsm, 350 mOsm, and 400 mOsm osmotic buffer contained additional 50 mM, 75 mM, 100 mM KCl, respectively, compared with 200 mOsm osmotic buffer.

### Measurement of intracellular ATP amount

Intracellular ATP amount was measured using “Cell” ATP Assay reagent Ver.2 (Wako, Cat#381-09306). HeLa cells were seeded in a 96-well white transparent bottom plate (Greiner Bio-One, Cat#655098 or CORNING, Cat#3610) and cultured for 24 h. 25 mM NaCl (control), 25 mM DCA, or 25 mM oxamate was pretreated 60 min before osmotic stress. Then, iso- or hyper-osmotic stress was applied for 15, 30, 60, and 120 min. After osmotic stimulation, all medium was removed and 50 μl per well of ATP reaction mixture (culture medium and assay reagent mixed in equal volumes) was added. Samples were incubated at room temperature for 10 min protected from light, and the luminescence was measured using Varioskan Flash. ATP amount at the timepoint of 0 min under isoosmotic conditions without inhibitors was normalized as 1.

### PI staining

HeLa cells were seeded in a 96-well plate and cultured for 24 h. 25 mM NaCl (control), 25 mM DCA, or 25 mM oxamate was pretreated 60 min before osmotic stress. Then, iso- or hyper-osmotic stress was applied for another 24 h. After osmotic stimulation, the cells were incubated with culture medium containing Hoechst 33342 (DOJINDO, Cat#346-07951) at a concentration of 1 μg/ml and PI (DOJINDO, Cat#343-07461) at a concentration of 0.3 μ g/ml for 30 min in 5% CO_2_ at 37 °C. The plate was measured and analyzed using the ArrayScan VTI with the optimized Cell Health Profiling BioApplication. Briefly, two image sets (Channel 1: nuclei stained with Hoechst 33342, Channel 2: PI) were acquired from nine fields per well. Cells were subsequently identified as targets from Channel 1, and a PI intensity was assigned to each target from Channel 2. Finally, cell death was calculated as the ratio of the number of PI-positive cells to the number of Hoechst-positive cells.

### Statistical analysis

All data are represented as the mean ± SD. Statistical tests was performed using Microsoft Excel or R software with RStudio. Unpaired two-tailed Student's *t* test (for two groups) and one-way ANOVA followed by Dunnett’s multiple comparisons test (for more than two groups) were used in this study. For all statistical analyses, ∗*p* < 0.05, ∗∗*p* < 0.01, ∗∗∗*p* < 0.001. *p* < 0.05 was considered statistically significant.

## Data availability

The authors declare that all data of our metabolome analysis and lipidome analysis supporting the findings of this study are available in supporting information (Tables S1 and S2).

## Supporting information

This article contains supporting information.

## Conflict of interest

The authors declare that they have no conflicts of interest with the contents of this article.
