# Supplementary material for: Mitochondria directly sense osmotic stress to trigger rapid metabolic remodeling via regulation of pyruvate dehydrogenase phosphorylation
Source: J Biol Chem. 2022 Dec 26;299(2):102837. doi: 10.1016/j.jbc.2022.102837 (PMC9879793; doi:10.1016/j.jbc.2022.102837)
Supplement: Supplemental Figures S1–S4 [file mmc1.docx]

**Supplementary Information**

Title

Mitochondria directly sense osmotic stress to trigger rapid metabolic remodeling via regulation of pyruvate dehydrogenase (PDH) phosphorylation

Authors

Takeshi Ikizawa^1^, Kazutaka Ikeda^2,3^, Makoto Arita^3,4^, Shojiro Kitajima^5^, Tomoyoshi Soga^5^, Hidenori Ichijo^1^*, and Isao Naguro^1^*

^1^Laboratory of Cell Signaling, Graduate School of Pharmaceutical Sciences, The University of Tokyo, 7-3-1 Hongo, Bunkyo-ku, Tokyo 113-0033, Japan

^2^Laboratory of Biomolecule Analysis, Department of Applied Genomics, Kazusa DNA Research Institute, 2-6-7 Kazusa-Kamatari, Kisarazu, Chiba, 292-0818, Japan

^3^RIKEN Center for Integrative Medical Sciences, 1-7-22 Suehiro-cho, Tsurumi-ku, Yokohama, Kanagawa 230-0045, Japan

^4^Division of Physiological Chemistry and Metabolism, Graduate School of Pharmaceutical Sciences, Keio University, 1-5-30 Shibakoen, Minato-ku, Tokyo 105-8512, Japan

^5^Institute for Advanced Biosciences, Keio University, Kakuganji, Tsuruoka, Yamagata 997-0052, Japan

*Correspondence: ichijo@mol.f.u-tokyo.ac.jp (H.I.), nagurois@mol.f.u-tokyo.ac.jp (I.N.)

**Supplementary Figures**

**
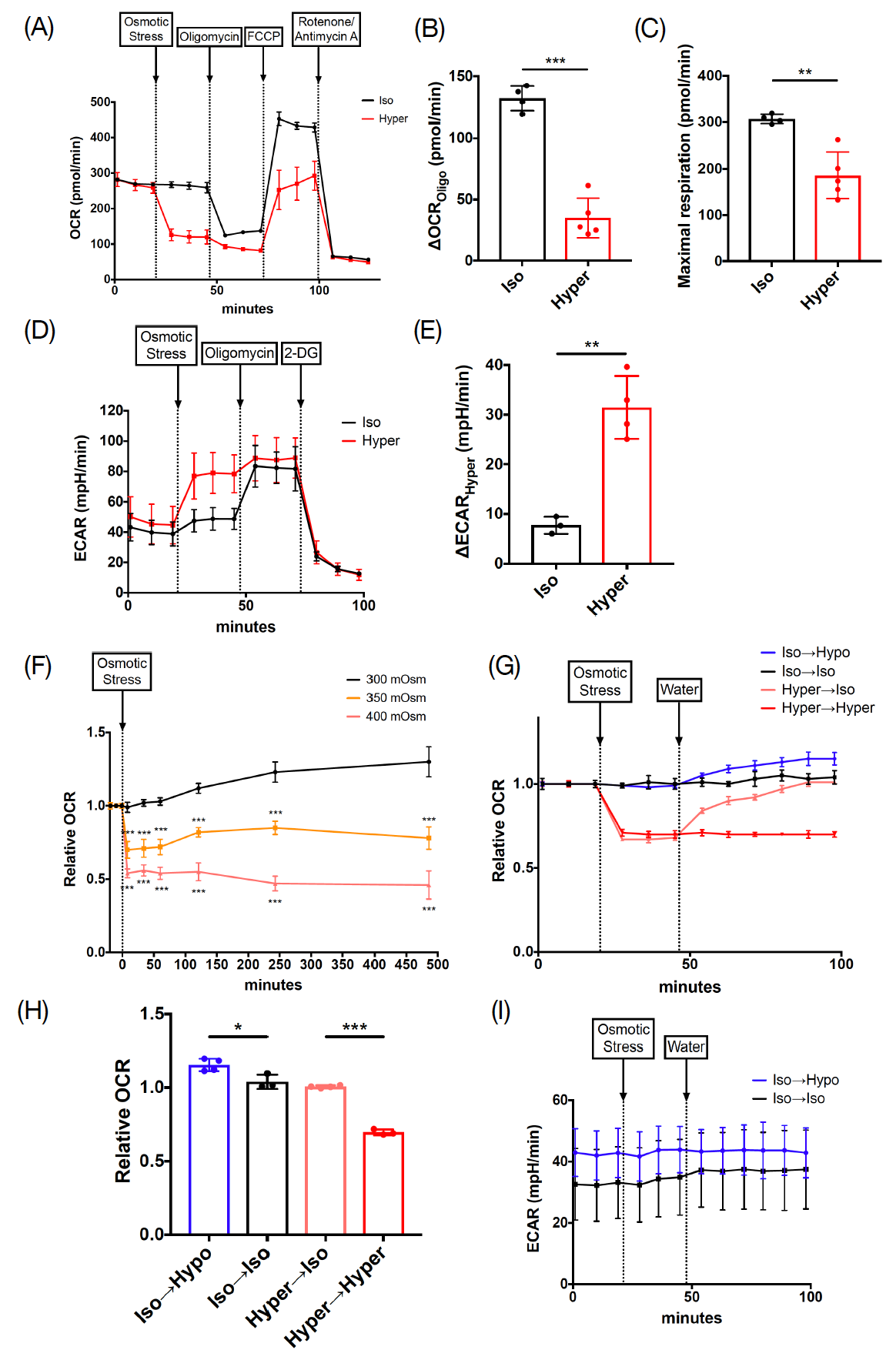
**

**Sup. Figure 1. Warburg-like metabolic remodeling is also induced by sorbitol-based hyperosmolarity, and its persistence, bidirectionality, and reversibility**

(A) OCR of HeLa cells was measured with sequential treatment with osmotic stress (by adding sorbitol), oligomycin, FCCP, and rotenone/antimycin A. A representative result from three independent experiments is shown. The trace was the average of 4-10 independent wells for each condition.

(B) ΔOCR_Oligo_ upon osmotic stress extracted from (A), which was calculated by subtraction between the average of three sequential measurements before and after oligomycin.

(C) Maximal respiration upon osmotic stress extracted from (A), which was calculated by subtraction between the average of three sequential measurements before and after FCCP.

(D) ECAR of HeLa cells was measured with sequential treatment with osmotic stress (by adding sorbitol), oligomycin, and 2-DG. A representative result from three independent experiments is shown. The trace was the average of 3-5 independent wells for each condition.

(E) ΔECAR_Hyper_ extracted from (D), which was calculated by subtraction between the average of three sequential measurements before and after osmotic stress.

(F) OCR of HeLa cells was measured until 8 hr after osmotic stress (by adding NaCl). The OCR is normalized by the average of the first three measurements. The timepoint of osmotic stimulation was indicated as 0 min. A representative result from three independent experiments is shown. The trace was the average of 6-7 independent wells for each condition. For simplicity, the measurements at about 8 min (the next measurement point after osmotic stress), 30 min, 1 hr, 2 hr, 4hr and 8 hr after osmotic stress are shown in the graph.

(G) OCR of HeLa cells was measured with the sequential treatment described below for each condition. Iso → Hypo: Medium → Water (1/3 volume of the medium, 225 mOsm finally), Iso → Iso: Medium → Medium, Hyper → Iso: 900 mOsm medium adjusted by NaCl (1/5 volume of the medium, 400 mOsm finally) → Water (1/3 volume of the medium, 300 mOsm finally), Hyper → Hyper: 900 mOsm medium adjusted by NaCl (1/5 volume of the medium, 400 mOsm finally) → 400 mOsm medium. The OCR is normalized by the average of the first three measurements. A representative result from three independent experiments is shown. The trace was the average of 3-5 independent wells for each condition.

(H) Values of relative OCR in the last measurement (98 min) extracted from (G).

(I) ECAR of HeLa cells was measured with the sequential treatment described below for each condition. Iso → Hypo: Medium → Water (1/3 volume of the medium, 225 mOsm finally), Iso → Iso: Medium → Medium. A representative result from three independent experiments is shown. The trace was the average of 3-5 independent wells for each condition.

Hypoosmotic stress: 225 mOsm, Isoosmotic stress: 300 mOsm, Hyperosmotic stress: 500 mOsm except (G) and (H). Data are represented as the mean ± SD. * p < 0.05, ** p < 0.01, *** p < 0.001. Unpaired two-tailed Student’s t-test.


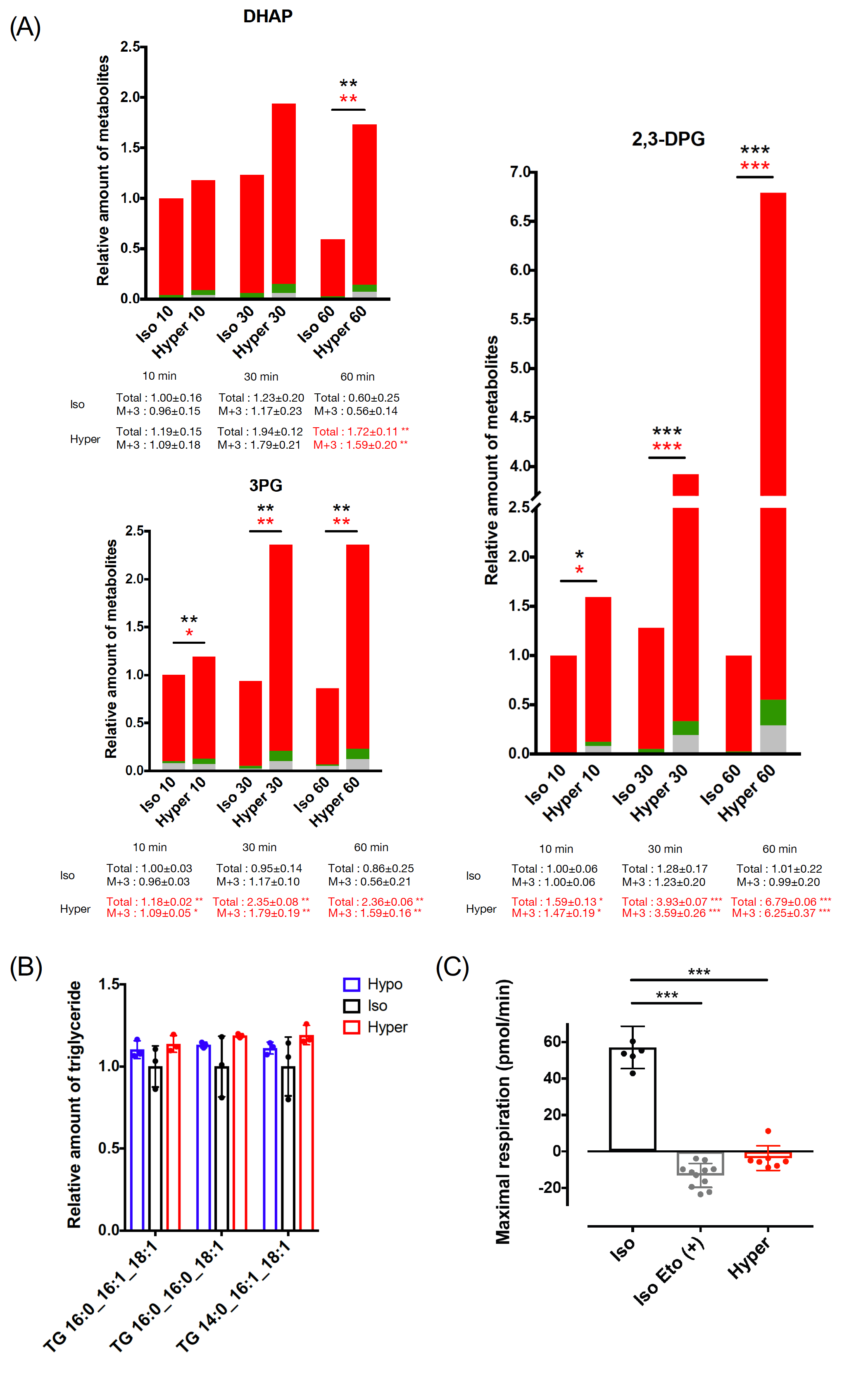


**Sup. Figure 2. Changes in metabolites under osmotic stress and suppression of FAO-dependent OCR under hyperosmotic stress**

(A) Related to Figure 2A, the relative amounts of metabolites in glycolysis (DHAP, 2,3-DPG and 3PG) are shown.

(B) Related to Figure 2D, relative amounts of several triglycerides composed of various combinations of fatty acids in HeLa cells at 15 min after osmotic stress. TG: triglyceride.

(C) Maximal respiration using the medium for measuring OCR dependent on fatty acid oxidation (see Methods), which was calculated by subtraction between the average of three sequential measurements before and after FCCP. A representative result from three independent experiments is shown. Three independent experiments were performed in which 5-11 independent wells were measured for each condition. Palmitate-BSA (150 µM) was added just before starting the assay. If present, Etomoxir (40 µM) was added 15 min before starting the assay.

Hypoosmotic stress: 200 mOsm, Isoosmotic stress: 300 mOsm, Hyperosmotic stress: 500 mOsm. Data are represented as the mean ± SD. N=3 (three samples for each condition) except (C). * p < 0.05, ** p < 0.01, *** p < 0.001. Unpaired two-tailed Student’s t-test at the same time points for (A). One-way ANOVA followed by Dunnett’s multiple comparisons test for (B), (C).

**
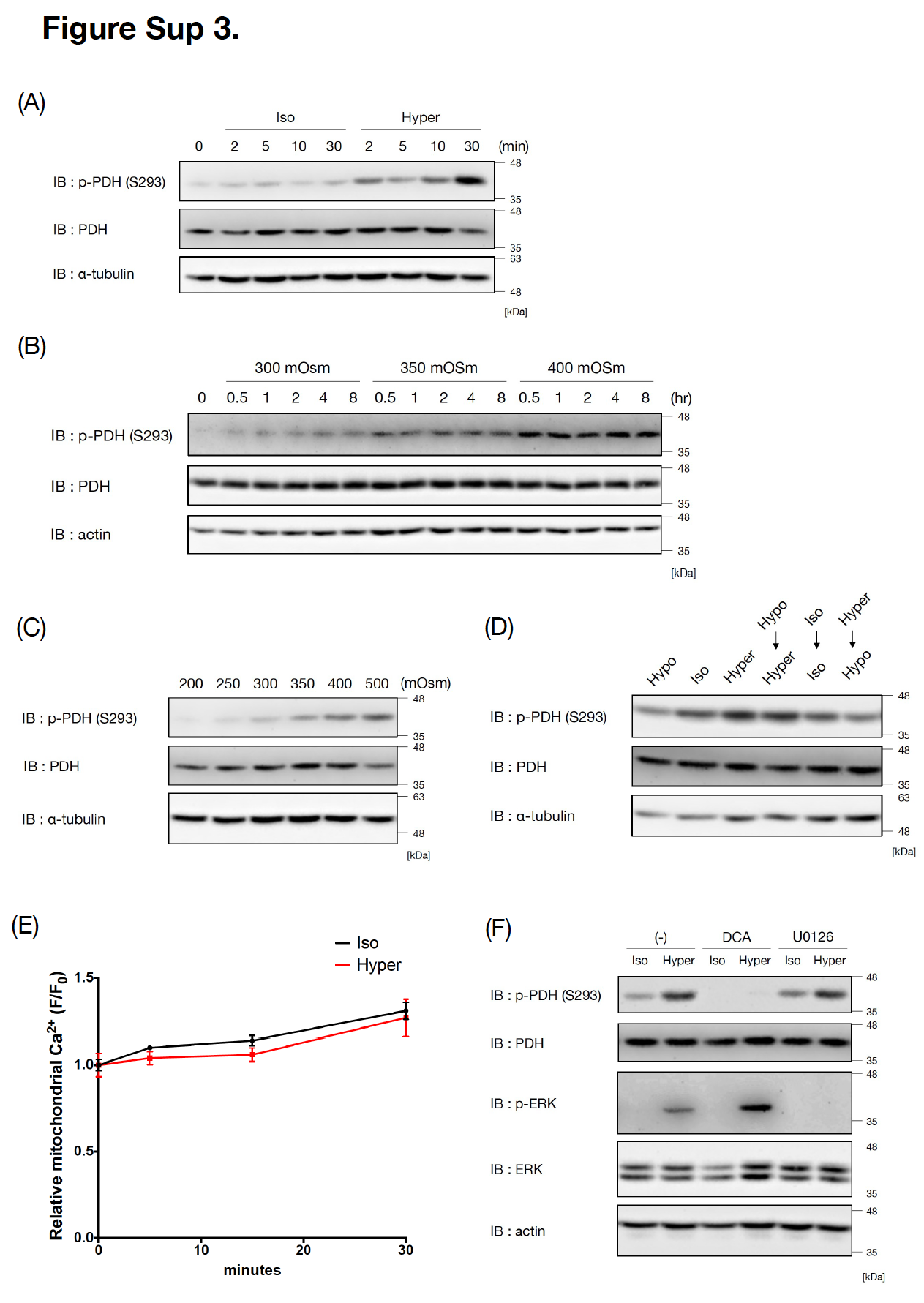

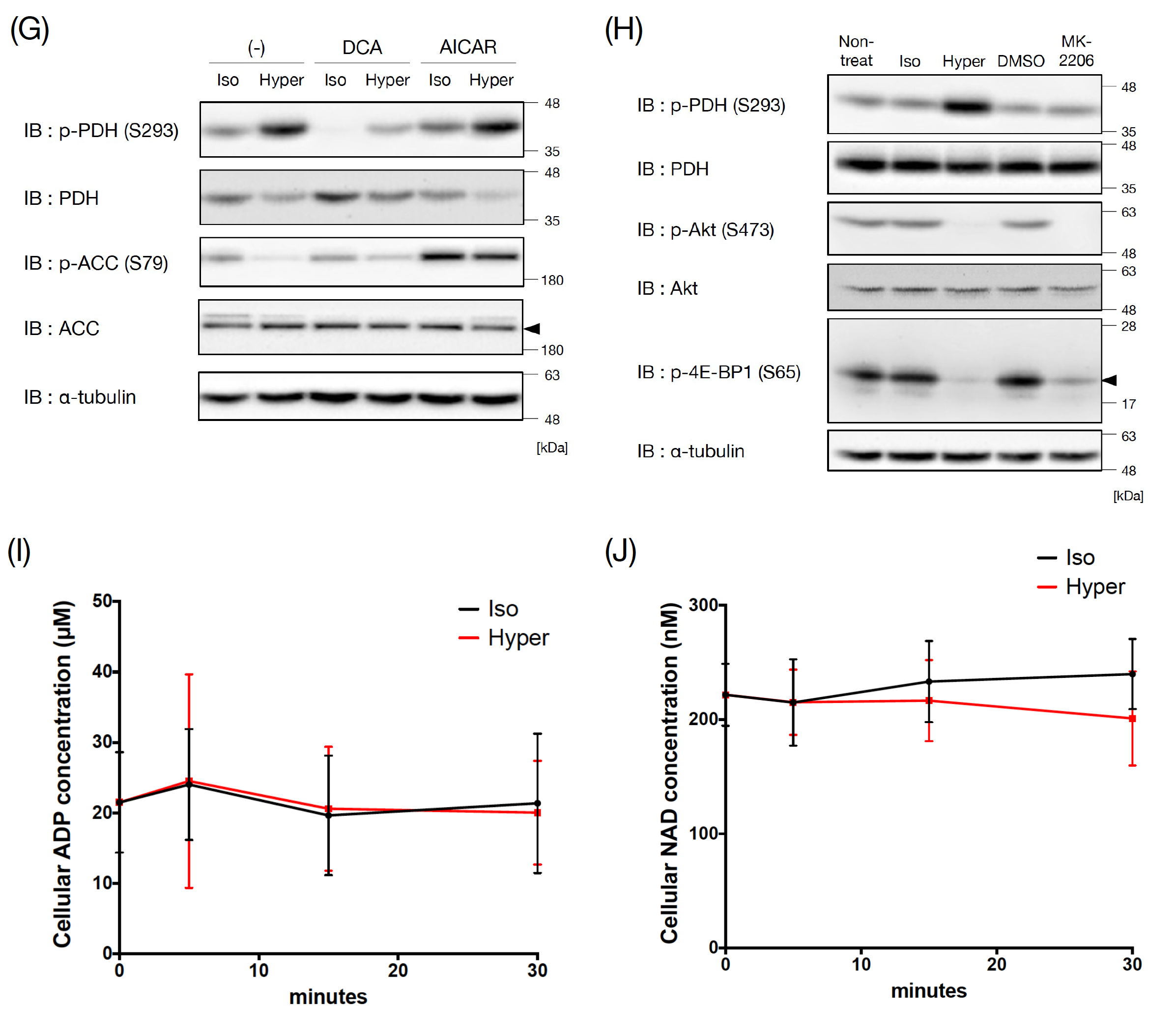
**

**Sup. Figure 3. Response of PDH phosphorylation under osmotic stress, and exploration of the upstream mechanism of the PDH regulation.**

(A) Immunoblot analysis of PDH phosphorylation after 2, 5, 10 and 30 min of osmotic stress by adding sorbitol to HeLa cells.

(B) Immunoblot analysis of PDH phosphorylation after 0.5, 1, 2, 4, 8 hr of osmotic stress by adding NaCl to HeLa cells.

(C) Immunoblot analysis of PDH phosphorylation under various osmolarities (Hypo: 200, 250, Iso: 300, Hyper: 400, 500 mOsm) by adding water or NaCl to HeLa cells. Osmotic stress: 30 min.

(D) Immunoblot analysis of PDH phosphorylation in HeLa cells when osmotic stress was changed halfway. Normal osmotic stress for 30 min in the left three lanes (Hypo: 200 mOsm, Iso: 300 mOsm, Hyper: 400 mOsm). In the right three lanes, osmotic stress was treated for 30 min, then changed as indicated for 60 min.

(E) Relative mitochondrial Ca^2+^ level in HeLa cells was measured after osmotic stress. The relative ratiometric fluorescence (F/F_0_) normalized by that at just before osmotic stimulation (time 0) is indicated. Three independent experiments were performed and averaged.

(F) Immunoblot analysis of PDH phosphorylation after 10 min of osmotic stress in HeLa cells pretreated with the MEK inhibitor U0126 (20 µM) or DCA (25 mM) for 60 min.

(G) Immunoblot analysis of PDH phosphorylation after 30 min of osmotic stress in HeLa cells pretreated with the AMPK activator AICAR (5 mM) or DCA (25 mM) for 60 min.

(H) Immunoblot analysis of PDH phosphorylation in HeLa cells after 10 min of osmotic stress and after treatment with the Akt inhibitor MK-2206 (500 nM) for 60 min under isoosmotic conditions.

(I) Cellular concentration of ADP in HeLa cells was measured after osmotic stress. The timepoint just before applying osmotic stimulation was indicated as 0 min. Three independent experiments were performed and averaged.

(J) Cellular concentration of NAD^+^ in HeLa cells was measured after osmotic stress. The timepoint just before applying osmotic stimulation was indicated as 0 min. Three independent experiments were performed and averaged.

Hypoosmotic stress: 200 mOsm, Isoosmotic stress: 300 mOsm, Hyperosmotic stress: 500 mOsm except (D). Data are represented as the mean ± SD.

**
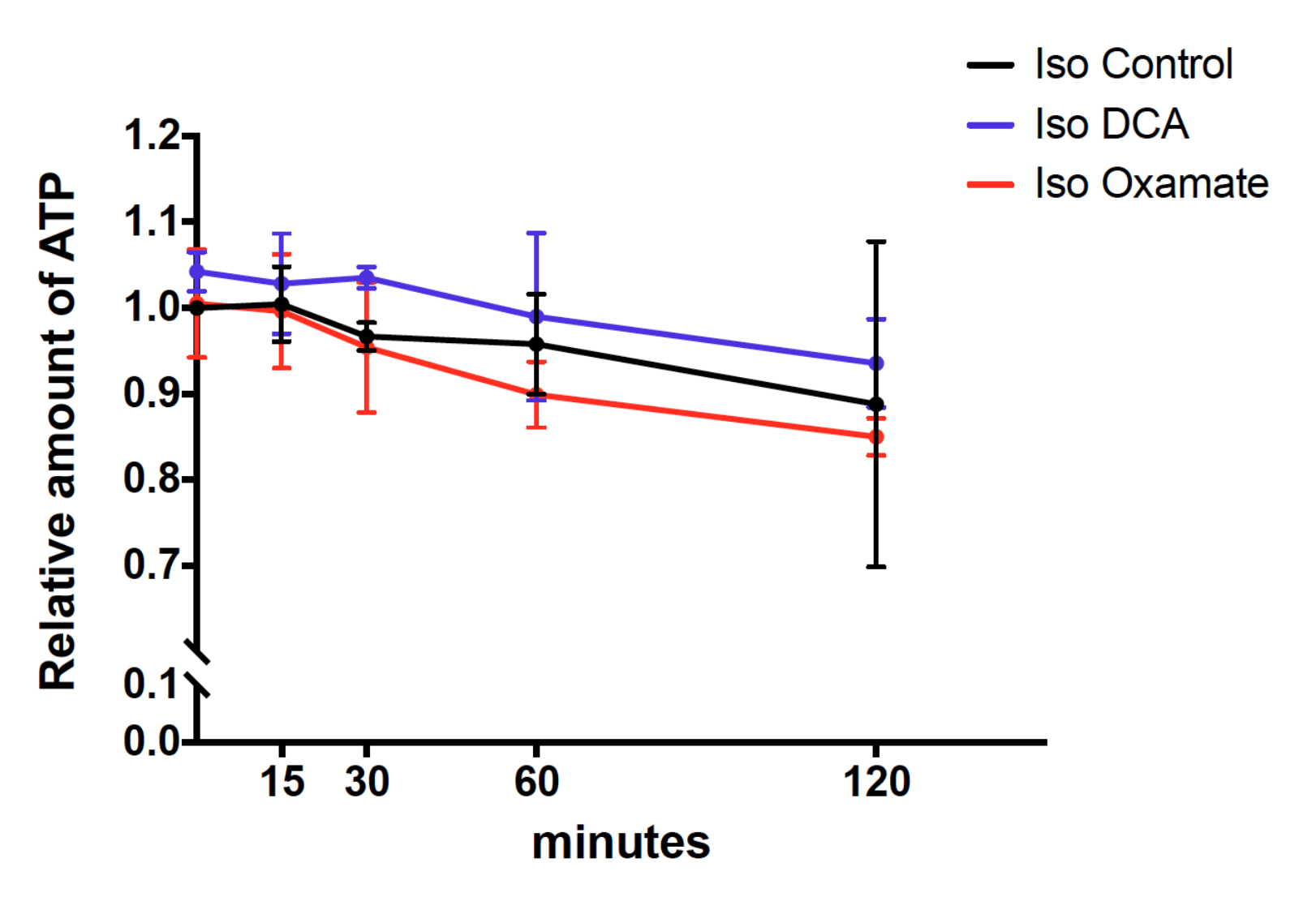
**

**Sup. Figure 4. Pretreatment with DCA or oxamate does not affect ATP levels under isoosmotic condition**

Related to Figure 4F, the relative amount of ATP in HeLa cells was measured under isoosmotic conditions with pretreatment with DCA (25 mM) or oxamate (25 mM) for 60 min using a plate reader. The timepoint just before applying osmotic stimulation was indicated as 0 min. Three independent experiments were performed and averaged. Isoosmotic stress: 300 mOsm. Data are represented as the mean ± SD.
